# Promoter-Dependent Translation Controlled by p54^nrb^ and hnRNPM during Myoblast Differentiation

**DOI:** 10.1371/journal.pone.0136466

**Published:** 2015-09-02

**Authors:** Nadera Ainaoui, Fransky Hantelys, Edith Renaud-Gabardos, Morgane Bunel, Frédéric Lopez, Françoise Pujol, Remi Planes, Elmostafa Bahraoui, Carole Pichereaux, Odile Burlet-Schiltz, Angelo Parini, Barbara Garmy-Susini, Anne-Catherine Prats

**Affiliations:** 1 TRADGENE, UPS (EA4554), Toulouse, France; 2 UMR U1037-CRCT, Inserm, UPS, Toulouse, France; 3 UMR U1043-CPTP, Inserm, UPS, Toulouse, France; 4 UMR U5282-IPBS, CNRS, UPS, Toulouse, France; 5 UMR U1048-I2MC, Inserm, UPS, Toulouse, France; Korea University, REPUBLIC OF KOREA

## Abstract

Fibroblast growth factor 1 (FGF1) is induced during myoblast differentiation at both transcriptional and translational levels. Here, we identify hnRNPM and p54^nrb^/NONO present in protein complexes bound to the FGF1 promoter and to the mRNA internal ribosome entry site (IRES). Knockdown or overexpression of these proteins indicate that they cooperate in activating IRES-dependent translation during myoblast differentiation, in a promoter-dependent manner. Importantly, mRNA transfection and promoter deletion experiments clearly demonstrate the impact of the FGF1 promoter on the activation of IRES-dependent translation via p54^nrb^ and hnRNPM. Accordingly, knockdown of either p54 or hnRNPM also blocks endogenous FGF1 induction and myotube formation, demonstrating the physiological relevance of this mechanism and the role of these two proteins in myogenesis. Our study demonstrates the cooperative function of hnRNPM and p54^nrb^ as regulators of IRES-dependent translation and indicates the involvement of a promoter-dependent mechanism.

## Introduction

Gene expression in eukaryotes is regulated at multiple levels. Transcription, as well as post-transcriptional processes such as mRNA splicing, polyadenylation, degradation and translation, require a wide range of multi-component cellular machines in order to finely control protein production. For a long time, these steps have been considered to be a simple linear assembly line. Then, it has become apparent that gene expression, including steps such as transcription, capping, splicing, polyadenylation, RNA export and degradation, is coordinated in a complex and extensively coupled network [1, 2]. However, coordination resulting from the co-transcriptional loading of mRNA processing proteins by the C-terminal domain of RNA polymerase II seemed to exclude translational regulatory complexes [3].

The fibroblast growth factor 1 (FGF1) gene provides an attractive system to decipher a mechanism of coupling between transcription and translation. Indeed, we have shown that this growth factor is induced by a transcription-translation coupling mechanism during myoblast differentiation [4]. The FGF1 gene structure has been well documented in human. Transcription occurs from four promoters A, B, C and D, which are either tissue specific or inducible [5, 6]. Promoters A, B and C are conserved in mouse [5]. The promoter A is active in heart, skeletal muscle and kidney while the promoter B is brain specific [4, 7]. Promoters C and D are inducible and considered as markers of cell proliferation [8–10]. The only FGF1 promoter to be activated during myoblast differentiation is the promoter A whereas the other promoters remain silent [4].

Transcription from each promoter generates alternative splicing of exon 1, leading to four mRNAs with distinct 5' untranslated regions (5'UTR), each of them expressing the same protein, FGF1. Two of these 5' UTRs contain internal ribosome entry sites (IRES) [11]. IRESs have been described in several mRNAs containing long structured 5' UTRs. Most of these mRNAs code for proteins involved in the control of gene expression [12]. IRES structures have been determined in several mRNAs by chemical and enzymatic methods, allowing identification of stem loops responsible for the IRES activity [11, 13, 14]. As regards the FGF1 IRES, the IRES structural domain is conserved in mammals [11]. However this IRES structure is different from the IRES structures found in other mRNAs. In particular, it clearly differs from the IRES structure of another member of the FGF family, FGF2, suggesting distinct regulations of these growth factors by the IRES-dependent mechanism [13]. IRES-mediated translation is involved in the major translational process in conditions when the classical mechanism of cap-dependent translation is blocked [14–16]. We have previously shown that FGF1 is translationally induced via the IRES present in the promoter A transcribed mRNA, while cap-dependent translation is blocked during the first steps of myoblast differentiation [4]. FGF1 IRES-mediated translation is concomitant with transcriptional activation at promoter A. Furthermore IRES activity is drastically enhanced when transcription is controlled by promoter A, compared to the cytomegalovirus (CMV) promoter [4].

The concept of a nuclear event governing formation of the “IRESome”, the ribonucleoprotein complex responsible for activation of IRES-dependent translation, has been reported in the literature for c-myc and Smad1 IRESs, although the cell factors responsible for this process have not been identified [17, 18]. Furthermore, most ITAFs are nuclear proteins primarily identified as splicing regulators. The first to be identified, pyrimidine tract binding protein (PTB), was described as an intron-binding protein before being characterized as an ITAF of encephalomyocarditis virus (EMCV) IRES [19, 20]. Several years after being identified as an ITAF for EMCV IRES, PTB has been shown to control several cellular IRESs such as APAF-1, BAG-1 or Bip [21–23]. Other splicing regulators, such as hnRNPK, hnRNPC1/2, hnRNPA1 or RBM4, have been identified as ITAFs [24–28]. HnRNPA1 has been described as an ITAF of several IRESs including FGF2, XIAP and c-myc IRESs [24, 29, 30]. It has been recently shown that this ITAF couples nuclear export and translation of several IRES-containing mRNAs [31].

Although the FGF1 IRES A has been reported to be stimulated during myoblast differentiation and muscle regeneration, nothing was known about ITAFs responsible for such regulation [4]. Biomolecular interaction analysis (BIA) uses a surface plasmon resonance phenomenon to characterize macromolecular interactions and has been recently optimized to enable the recovery and identification of interacting molecules by coupling BIA with mass spectrometry (MS) [32]. BIA-MS has proven successful in the identification of the partners in protein-protein interactions as it provides a high sensitivity compared to classical affinity chromatography approaches and permits identification from small quantities of bound ligands. A very reproducible "microelution" is now possible with the BIACORE 3000 instruments. This novel technology was selected in the present study to search for proteins bound to the FGF1 promoter A and IRES A during myoblast differentiation to find candidate ITAFs and transcription factors involved in the transcription-translation coupling mechanism.

In the present report, we identified hnRNPM and p54^nrb^/NONO bound (directly or indirectly) to FGF1 promoter A and IRES A in differentiating myoblasts (C2C12). Knockdown and overexpression approaches revealed that p54^nrb^ and hnRNPM are required to activate the IRES-dependent translation in a promoter-dependent manner. Furthermore, p54^nrb^ and/or hnRNPM knockdown inhibited myotube formation. Altogether, this study identifies a novel regulation of FGF1 gene expression, implying a cooperation between promoter and translational regulators to promote the time controlled process of myoblast differentiation.

## Materials and Methods

### Plasmids

The bicistronic plasmids with the CMV promoter and FGF1 IRES A (pCRF1AL2) or EMCV IRES (pCREL2), with the FGF1 promoter A and IRES A (pP1ARF1AL2) were previously described [4]. Human hnRNPM4 and p54^nrb^ cDNAs were the gifts of M. Swanson and A. Krainer, respectively. cDNAs were PCR-amplified and introduced into the pTRIP-DU3-MCS vector (pTRIP-p54 and pTRIP-HM) [33–35]. The bicistronic vector pTRIP-p54iHM contains the two cDNAs separated by the FGF1 IRES. Plasmid construction details are available upon request.

### Cell culture and transfection

C2C12 myoblasts (European Collection of Cell Culture ECACC No 91031101) were maintained in Dulbecco’s modified Eagle’s medium (DMEM) with 20% fetal calf serum in 100-mm diameter dishes at 37°C with 5% CO2. For differentiation, cells were changed into fusion medium (DMEM with 5% horse serum). Transient transfections were performed in 6-well dishes using 1 μg plasmid with FuGene-6 (Biochemichals) and OptiMEM (Gibco-BRL, Invitrogen).

Small interference RNAs were from Thermoscientific ONE TARGET plus SMARTpool targeting hnRNP M (siM) cat L-013452-01 lot 100508 or p54 cat L-007756-01 lot 100809 (sip54) or siGENOME non-targeting siRNA (sic). Only in S3 File, different siRNAs were used: siM-2 (5’-CAUUGGAAUGGGAAACCUATT-3’) and sip54-2 (5’-GCUGAAUUUGCUCCAAAUATT-3’) (Sigma). C2C12 cells were transfected with 50nM siRNA with Hyperfect transfection reagent (Qiagen). mRNA transfection, were performed using Lipofectamine 2000 (Invitrogen) according to the manufacter’s recommendations, with 2μg of each bicistronic mRNA. Cells were incubated at 37°C for 12h before harvesting and analysis.

### Cell fractionation

C2C12 cells on 14 cm culture dishes were rinsed twice in cold phosphate-buffered saline (PBS 1X). Cells were scraped with a rubber policeman in 1 ml PBS 1X and pelleted by centrifugation at 2500 g for 10 min at 4°C. The pellet was resuspended in the 400 μl buffer A (10 mM HEPES pH7.9; 10 mM KCl; 0,1 mM EGTA; 1 mM DTT; 1X proteases inhibitors). The cells were allowed to swell on ice for 15 min. 25 μl the buffer B (100 mM HEPES; 100 mM KCL2; 10 mM EGTA; 500 mM MgCL2; 10% NP40) was added and the tube was vigorously vortexed for 10 sec. The homogenate was centrifuged for 30 sec; the supernatant containing cytoplasmic extract was recovered. The nuclear pellet was resuspended in 50 μl the ice-cold buffer C (20 mM HEPES, 400 mM NaCl; 1mM EGTA; 1 mM DTT; 1% proteases inhibitors) and vigorously rocked at 4°C for 15 min on a shaking platform. The nuclear extract was centrifuged for 5 min at 4°C and the supernatant recovered.

### Preparation of biotinylated RNA and DNA, and of capped and polyadenylated mRNA

The FGF1 5’UTR cDNA containing the IRES A sequence obtained by PCR was cloned in the pCR 4Blunt-TOPO plasmid (Invitrogen) downstream the T3 sequence [11]. The IRES T3 promoter fragment was cut with NcoI and transcription was performed with the MEGAscript T3 (Ambion), as per the manufacturer’s protocol, in the presence of UTP-16-biotin (Roche Diagnostics GmbH), and the newly synthesized RNA was purified using an RNeasy column (Qiagen). The FGF1 promoter A was amplified from the bicistronic plasmid pP1AF1AL2 with the 5’ biotin primers purchased from Invitrogen [4]: primer 5’biotin forward: 5'agcttaggtgaggagcctttcca-3', reverse: 5'accctgaaaggcagatgtgg-3'.

The purification PCR products were performed by the Nucleospin Extract II kit (Macherey-Nagel).

Capped and polyadenylated bicistronic mRNAs containing the FGF-1 IRES-A or the EMCV IRES were obtained using as templates PCR fragments amplified from bicistronic plasmids pCRF1AL2 and pCREL2, respectively, using primers (oligo(dt) included in the 3’ primer) purchased from Sigma [4]. Transcription in vitro was performed with mMessage mMachine T3 Ultra kit (Ambion) according to the manufacter’s recommendations.

### BIA-MS experiments

SPR experiments were performed on a Proteon XPR36 (Biorad) and BIAcore 3000 (GE Healthcare) apparatus. The biotinylated targets RNA and DNA were immobilized on NLC sensorchip (Biorad) or carboxymethylated dextran sensor chips coated with streptavidin (SA sensorchip BIAcore) prepared according to the manufacturer’s instructions. All RNA and DNA samples were prepared in HBS-EP buffer (0.01 M HEPES, pH 7.4; 15 M NaCl; 3 mM EDTA; 0.005% 20 surfactant). The proteins were injected at 20 μl/min at 20°C, at 500 μg/mL concentration across the sensor surface in this buffer. The regeneration of the RNA or DNA coated surface was achieved at 90 sec with a solution (20mM TEA; 0.5 M Urea).

Eluted protein samples (6 μL) were digested by the addition of 10μL of a solution of modified trypsin in 25 mM NH_4_HCO_3_ (20 ng/μL, sequence grade, Promega) at 37°C for 4h. The peptides mixtures were analyzed by nanoLC-MS/MS using a nanochromatography system (Ulmitate, Dionex) coupled to a Q-Star (Applied Biosystems, Altringham, USA) or an LTQ-Orbitrap (Thermo Fisher Scientific) mass spectrometer. Five microliters of each sample were loaded on a C18 precolumn (300 μm inner diameter x5 mm; Dionex) at 20 μL/min in 5% acetonitrile, 0.05% trifluoroacetic acid. After 5 min of desalting, the precolumn was switched on line with the analytical C18 column (75-μm inner diameter x 15 cm; PepMap C18, Dionex) equilibrated in 95% solvent A (5% acetonitrile, 0.2% formic acid) and 5% solvent B (80% acetonitrile, 0.2% formic acid). Peptides were eluted using a 5–50% gradient of solvent B during 80 min at a 300 nl/min flow rate. The mass spectrometer was operated in data-dependent acquisition mode. MS spectra were acquired on the 300–2000 *m*/*z* range and the three most intense ions were then selected for CID fragmentation. Dynamic exclusion was used within 60 s to prevent repetitive selection of the same peptide.

### Database search and data analysis

The Mascot Daemon software (version 2.3.2, Matrix Science, London, UK) was used to perform database searches in batch mode with all the raw files acquired on each sample. Data were searched against all entries in the SwissProt Human Mouse_20090127 protein database (36322 sequences; 19974433 residues). Oxidation of methionine was set as a variable modification for all Mascot searches. Specificity of trypsin digestion was set for cleavage after Lys or Arg except before Pro, and one missed trypsin cleavage site was allowed. The mass tolerances in MS and MS/MS were set to 5 ppm and 0.6 Da, respectively. Mascot results were parsed with the in-house developed software Mascot File Parsing and Quantification (MFPaQ) version 4.0 and protein hits were automatically validated if they satisfied a false discovery rate (FDR) of 1% with peptides of a minimal length of 8 amino acids [36].

### ChIP

C2C12 cells on 14 cm culture dishes (10 millions cells per dish) were treated 10 min at 25°C with cross-linking buffer (buffer A, ChIP Diagenode kit) at a final concentration of 1% formaldehyde. The cross-linking reaction was quenched by addition of glycine at 0.125 M (5 min at 25°C) and the cross-linked cells were washed with the ice-cold PBS and treated with 500μl of lysis buffer (buffer B, Diagenode kit). Lysates were centrifuged for 5 min at 4°C, pellets were resuspended in 50 μL of buffer C containing protease inhibitors and incubated for 10 min at 4°C with gentle mixing. DNA was adjusted at a concentration of 10ng/μL as recommended by the supplier. The chromatin samples were sonicated for 15 cycles of 30 sec “ON”/30 sec “OFF” using the Bioruptor from Diagenode, then subjected to immunoprecipitation using antibodies anti-hnRNPM (1/D8) (Santa Cruz Biotechnologies), or anti-p54^nrb^/NONO (BD Bio-sciences), or without antibody for the “mock” control. The immunoprecipitates or the input without immunoprecipitation were qPCR-amplified using according to the manufacturer's instructions. The TBP (TATA binding protein) gene was used as a reference gene.

### RNA immunoprecipitation (RIP)

RNA-binding protein immunoprecipitation (RIP) is the RNA analog of the more well-known ChIP application and used to identify specific RNA molecules (of many types) associated with specific nuclear or cytoplasmic binding proteins. C2C12 cells on 14 cm culture dishes were treated for 10 min at 25°C with cross-linking buffer containing formaldehyde at 1% final concentration (buffer A, Magna RIP kit from Millipore). The cross-linking reaction was quenched for 5 min at 25°C with addition of glycine to 0.125 M. The cross-linked cells were rinsed twice in cold phosphate-buffered saline (PBS 1X). Cells were scraped with a rubber policeman in 10 mL PBS 1X and pelleted by centrifugation at 1500g for 5 min at 4°C. Pellets were resuspended in 500 μL of RIP lysis buffer (1% NP40, 10 mM HEPES, 100 mM KCl, 5 mM MgCl_2_, protease inhibitor cocktail 1x, RNases inhibitors, Magna RIP kit, Millipore).

50 μL of magnetic beads protein A/G was washed four times in 500μL of RIP wash buffer (0.05M Tris, 0.15M NaCl, pH 7.5) then incubated for 30 min at room temperature with 10μg of antibody anti-hnRNPM (1D8 Santa Cruz Biotechnologies), anti-p54^nrb^/NONO (BD Bio-sciences) or anti-IgG. The beads-antibody complex was washed twelve times in 500 μL of RIP wash buffer before adding 900 μL of immunoprecipitation buffer (RIP wash buffer: 0.5 M EDTA, 5 μL RNase inhibitor). The cell lysate was centrifuged at 14000 rpm for 10 min at 4°C, 100 μL of the supernatant was added to each sample of beads-antibody complex and incubated at 4°C overnight. Immunoprecipitates were washed six times before protein and RNA recovery. Proteins were recovered with 50 μl of SDS-PAGE loading buffer and analyzed by Western Blot. RNA was recovered by incubation of the immunoprecipitate for 30 min at 55°C in 150 μl of proteinase K buffer containing 117 μL of RIP wash buffer, 15 μL of SDS 10% and 18 μL of proteinase K at 10 mg/mL. The immunoprecipitates were analyzed by RT-PCR using the High Capacity cDNA Reverse Transcription kit from Applied Biosystems and qPCR-amplified according to the manufacturer’s instructions.

### Immunoprecipitation

C2C12 cells were harvested in 1 mL PBS (1X), pelleted and resuspended in 100μl of lysis buffer (RIPA). 100 μL Protein G-plus or Protein A was precoated with mouse anti-hnRNPM (1D8 Santa Cruz Biotechnologies) or anti-p54 antibody (BD Biosciences), and was incubated with 70 μL of the cell lysate. After 5 times washing with IP buffer plus 0.5% BSA, proteins were eluted in 100 μL elution buffer and 50μL was loaded on SDS-PAGE gel for Western analysis using rabbit antibodies against hnRNPM (Sigma AV40620), p54 (Sigma N8789). Mock experiments were performed similarly but without primary antibody.

### Western blotting

C2C12 were harvested in lysis buffer (50 mM Tris-HCL pH8, 150mM NaCl, 1mM EDTA, 1% NP40, 20 μL protease inhibitor mixture (Roche) and clarified by centrifugation at 13000 rpm for 10 min at 4°C. Proteins supernatant were quantified using the Bradford method (Biorad).

Primary antibodies were mouse monoclonal antibodies against hnRNPM (Santa Cruz Biotechnologies, 1/400), p54nrb (BD Bio-sciences, 1/400), myogenin (Santa Cruz Biotechnologies 1/400), Glyceraldehyde 3-phosphate deshydrogenase (GAPDH, Santa Cruz biotechnologies 1/10000) and the rabbit polyclonal FGF1 (F5521, sigma, 1/400). Secondary antibodies were peroxidase-conjugated-AffiniPure Donkey Anti-Rabbit IgG and AffiniPure Donkey Anti-Mouse IgG (Jackson ImmunoResearch, 1/10000).

Protein detection was carried out using Supersignal West Pico Cheluminescent Substrat and ECL western blotting substrate (ThermoScientific).

### RNA extraction and real time RT-qPCR

Total RNA was isolated from C2C12 cells using Nucleospin RNA II kit (Macherey-Nagel). After DNase I treatment (DNase I Amplification Grade, Invitrogen), reverse transcription was performed with the High capacity cDNA Archive Kit (Applied Biosystems, Foster City, CA). As an internal control, ribosomal 18S (m18S) RNA was used.

Quantitative PCR was performed on a Stepone sequence detection system (Applied Biosystems) using Sybr Green PCR Master Mix (Applied Biosystems) for detection of LucF, LucR, FGF1 and 18S transcripts.

Primer sequences are available upon request.

### Reporter activity assay

Protein from C2C12 cells were extracted with reporter Passive Lysis buffer (Promega) and protein concentration was quantified by Bradford Standard Assay. Quantification of bioluminescence was performed with a luminometer (Centro LB960, Berthold) using the Dual-Luciferase Reporter Assay (Promega France).

### Ethics statements

Experiments have been conducted with the genetically modified organisms agreement n°496 from the French Technologies High Committee.

Authors comply with best practices in publication ethics, specifically regarding authorship, dual publication, plagiarism, figure manipulation, and competing interests.

## Results

### BIA-MS identification of p54^nrb^ and hnRNPM bound to the FGF1 promoter and IRES

**Fig 1:**
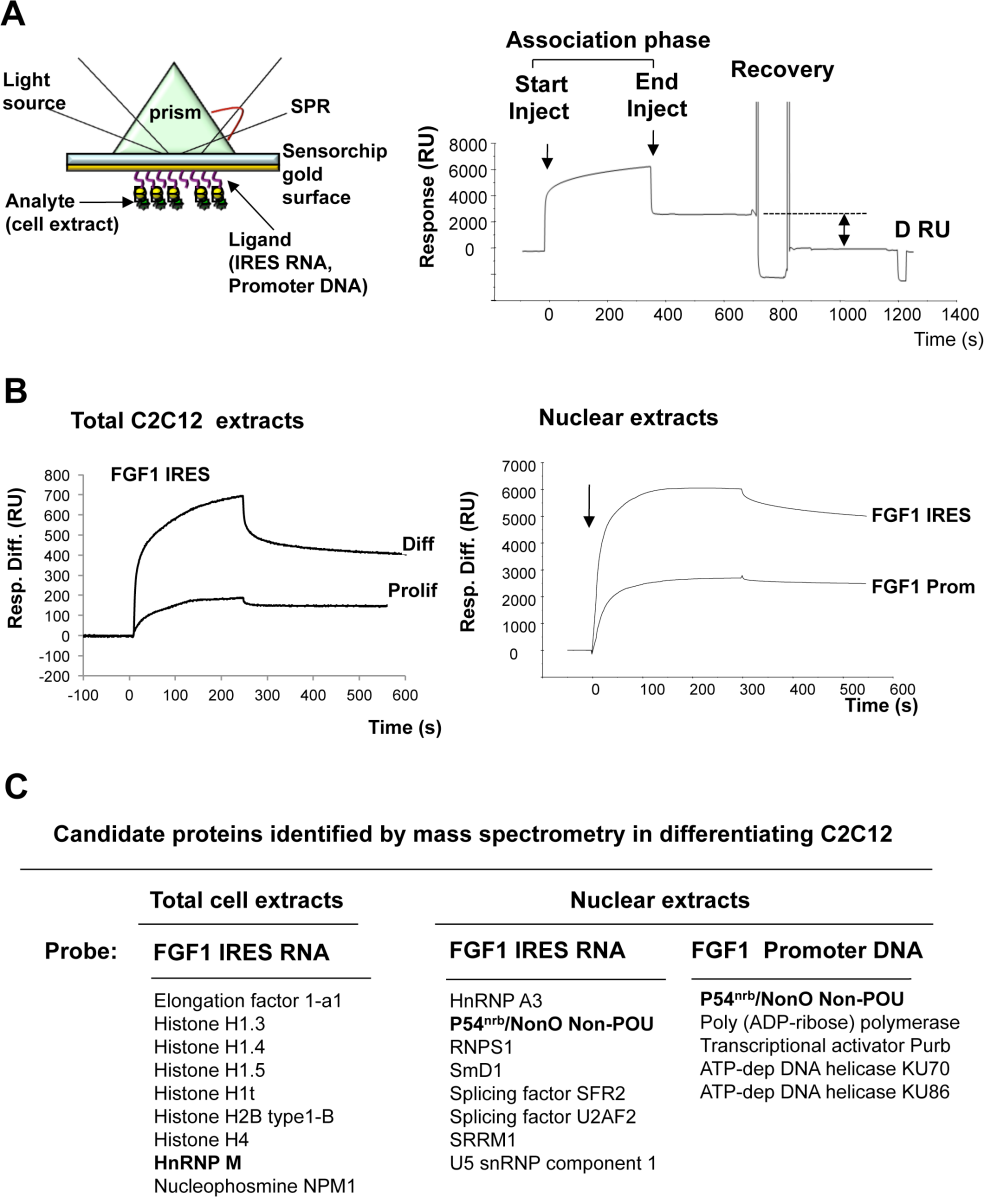
Identification of RNA and DNA binding proteins by BIA-MS. (A) BIACORE 3000 analysis using surface plasmon resonance. Left: the ligand corresponding to biotinylated RNA or DNA is immobilized by streptavidin coupled to the sensorchip gold surface. On the other side of the sensorchip, a light source is directed to the sensorchip through a prism and is reflected in all angles except one at which it is absorbed into the gold surface in the form of an evanescent wave. The binding of the analyte corresponding to RNA or DNA binding proteins to the ligand results in a change in the index of refraction proportional to the number of bound molecules. This generates a shift in the absorption angle that is recorded by the detector and appears on a sensogram. Right: Sensogram generated during a binding cycle followed by protein recovery. The response appears in resonance units (RU). The association phase lasts from the start to the end of analyte injection (400 s). Protein recovery is achieved at 800 s and the ΔRU indicates the efficiency of dissociation of the bound proteins. (B) C2C12 myoblast protein binding and recovery. Total extracts of proliferating or differentiating C2C12 myoblasts (left) or nuclear extracts of differentiating C2C12 (right) were injected as analytes in several BIACORE 3000 channels after immobilization of ligands corresponding to FGF1 IRES A RNA (nt 1 to 442) or promoter A DNA (distal part nt 1 to 391). Bound proteins were recovered as described in Mat. & Meth. (C) Mass spectrometry analysis of proteins recovered from the BIACORE 3000 experiments. For each BIA-MS experiment, 6 recovery cycles were pooled to obtain a sufficient RU quantity (about 2000 RU). The mass spectrometry analysis was performed as described in Mat. & Meth. The most significant proteins (RNA and DNA binding proteins) are listed here, whereas the complete list is provided in S1 File. hnRNPM and p54^nrb^ have been selected as the most interesting candidates.

BIA-MS technology was used to identify proteins bound to the FGF1 promoter A and IRES A. Biotinylated promoter DNA and IRES RNA were (separately) immobilized on biacore streptavidin sensorchips (Fig 1A). This method is not denaturing and allows identification of proteins present in the complex bound to DNA or RNA. Binding to nucleic acids may be direct or indirect. Total or nuclear cell extracts, from proliferating or differentiating C2C12 cells (day 2), were injected into the BIACORE 3000 apparatus to obtain the association phase (Fig 1B). Interestingly, a significant association was obtained with extracts of differentiating cells, but not with proliferating cell extracts. Using nuclear extracts an important binding activity was obtained with both FGF1 IRES RNA and promoter DNA. Bound proteins were recovered and identified by nanoLC-MS/MS after tryptic digestion (Fig 1C and S1 File). Proteins from total extracts bound to the IRES included: 1) ribosomal proteins and elongation factor eEF1A1, expected to be involved in translation and 2) nuclear proteins including several histones, nucleophosmin, and the splicing protein hnRNPM (Fig 1C, Table A in S1 File) [37–39]. Using nuclear extracts, we identified IRES interactions with the splicing proteins hnRNPA3, U2AF2, SFR2 and U5 snRNP component S1 as well as p54^nrb^, a protein involved in both splicing and transcription (Fig 1C, Table B in S1 File) [27, 28]. Interestingly, none of these proteins bound the EMCV IRES, used as a control (Table C in S1 File). Several DNA binding proteins were identified as interacting (directly or indirectly) with the FGF1 promoter: Poly(ADP-ribose)polymerase (PARP), transcriptional activator Purβ, ATP-dependent DNA helicases KU70 and KU86 and p54^nrb^, which was also bound to the IRES (Fig 1C, Table D in S1 File). The dual presence of p54^nrb^ promoted it as the most interesting candidate. Furthermore, its interaction with hnRNPM and co-localization within defined nuclear structures incited us to focus on the role of these two proteins in the control of FGF1 expression during myoblast differentiation [40].

### P54^nrb^ and hnRNPM bind to FGF1 promoter and IRES in differentiating, but not in proliferating myoblasts

**Fig 2:**
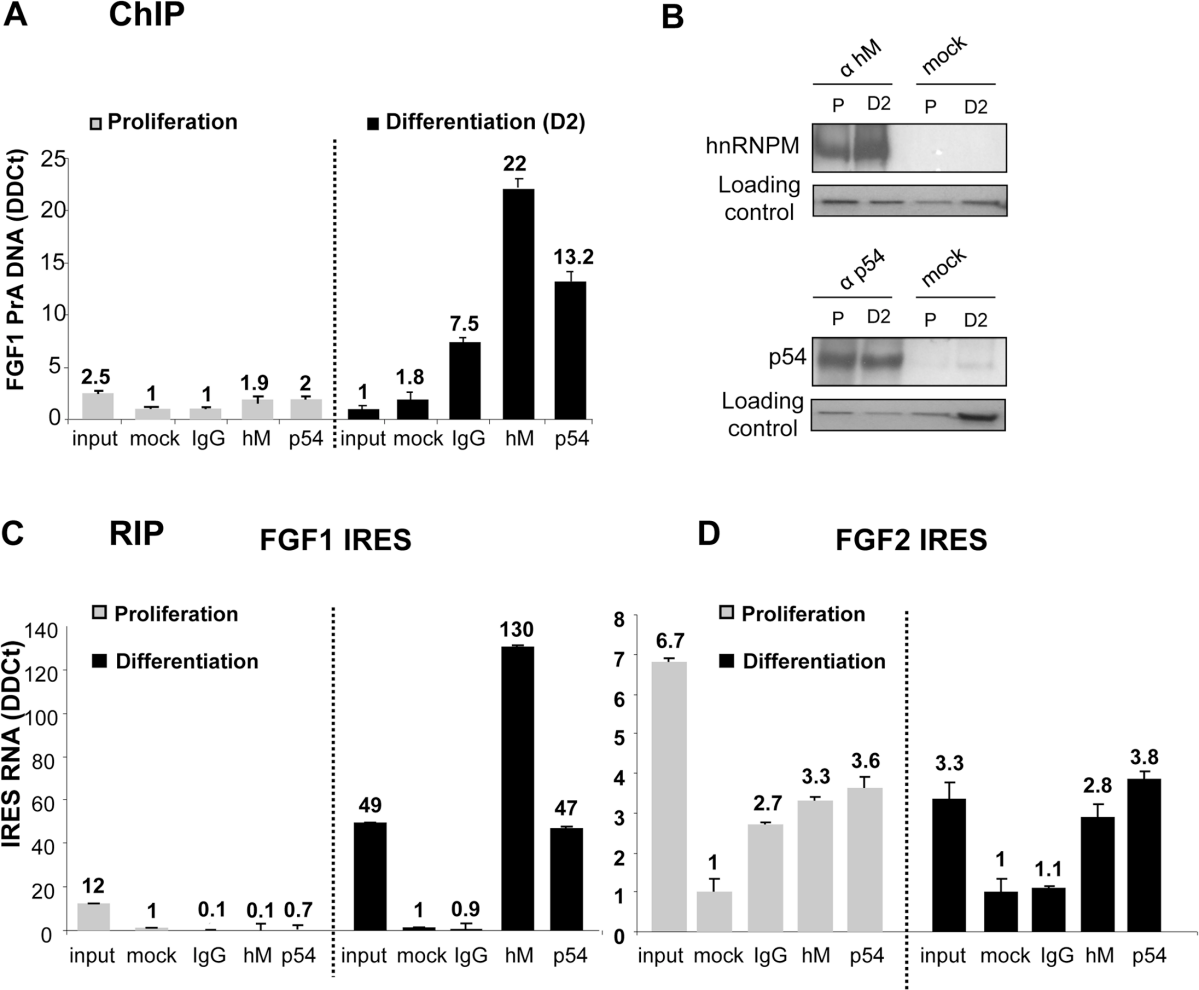
Interaction of hnRNPM and p54^nrb^ proteins with FGF1 promoter and IRES. (A) ChIP assays were performed as described in Mat. & Meth. with genomic DNA purified and fragmented from C2C12 extracts in proliferation (left panel) or differentiation 2 days after serum-starvation treatment (right panel). Immunoprecipitation experiments were performed with anti-hnRNPM (1/D8), anti-p54^nrb^ antibodies, control IgG provided by the manufacturer (IgG) or without antibodies (mock), respectively. qPCR quantification was achieved with primers specific to the 1–391 FGF1-A promoter fragment or with primers specific to the TBP gene used as the reference gene. The values are expressed relatively to the reference gene. Experiments were performed in biological triplicates and repeated three times. A representative experiment is shown (mean +- standard deviation). (B) HnRNPM and p54^nrb^ protein levels were analyzed by immunoprecipitation followed by Western blotting in proliferating (P) or differentiating (day 2 after serum-starvation, D2) C2C12 cell lysates, using anti-hnRNPM or antip54 antibodies. The loading control was checked by Ponceau Red staining. (C, D) RIP assays were performed as described in Mat. & Meth. with cell lysates from proliferating or differentiating myoblasts. Immunoprecipitation was achieved as in (A). RNAs present in the immunoprecipitated complexes were quantified by RT qPCR using primers specific to the FGF1 (C) or FGF2 IRES (D). The reference gene was 18S RNA, reflecting non specific RNA binding. The values are expressed relatively to the control (mock) without antibody. Experiments were performed in biological triplicates and repeated three times. A representative experiment is shown (mean +- standard deviation).

Interaction of hnRNPM and p54^nrb^ with the promoter was analyzed by chromatin immunoprecipitation (ChIP), using DNA extracts either from proliferating or differentiating myoblasts (Fig 2A). We checked by immunoprecipitation that the two proteins are expressed in proliferating as well as in differentiating cells (Fig 2B). However no interaction was detected with either of the two proteins in proliferating cells, whereas significant interaction was observed with both proteins in differentiating myoblasts (Fig 2A). RNA immunoprecipitation (RIP) was also performed using anti-hnRNPM or anti-p54 antibodies revealing that the two proteins interact with the FGF1 IRES RNA in differentiating, but not in proliferating, myoblasts (Fig 2C). These data demonstrated that hnRNPM and p54^nrb^ interact (directly or indirectly) with FGF1 promoter A and IRES A only in differentiating myoblasts. In contrast the two proteins very poorly interact with the FGF2 IRES, and such interaction did not increase during differentiation (Fig 2D).

Thus, hnRNPM and p54^nrb^ binding to FGF1 promoter and IRES correlates with the previously shown induction during differentiation [4]. Binding and activity are very weak during proliferation and induced by day 2 of differentiation. This suggests that these proteins may be involved in the specific activation of FGF1 mRNA accumulation and IRES-mediated translation.

### p54^nrb^ and hnRNPM knockdown silences the FGF1 promoter-dependent accumulation of mRNA during myoblast differentiation

**Fig 3:**
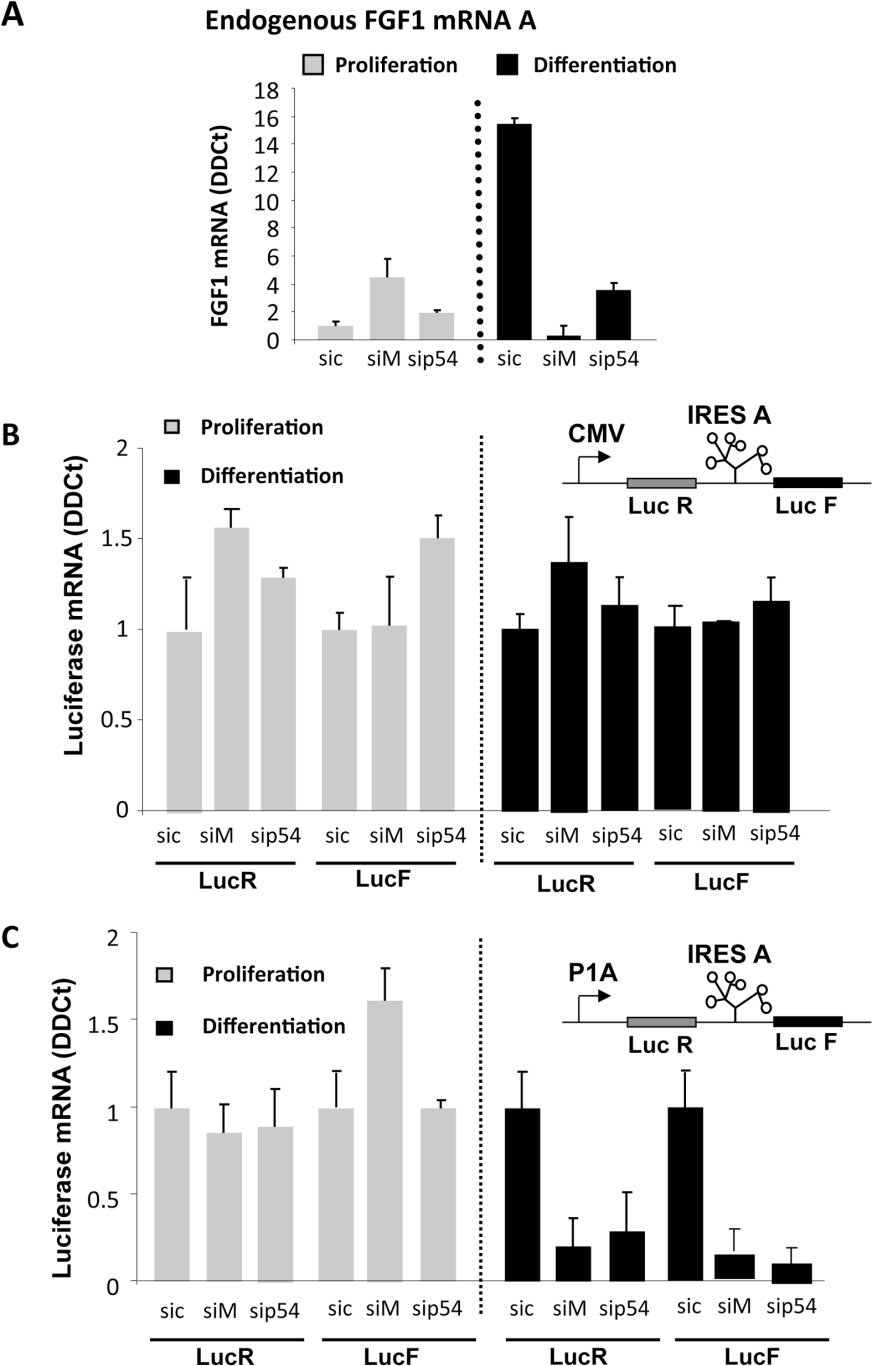
Effect of hnRNPM and p54^nrb^ knockdown on expression of endogenous FGF1 mRNA and bicistronic mRNAs during myoblast differentiation. (A) RT qPCR quantification of endogenous FGF1 mRNA A was performed as described in Mat. & Meth. during C2C12 myoblast proliferation or day 2 differentiation after transfection with siRNA ONE TARGET PLUS smartpool against hnRNPM (siM), p54^nrb^ (sip54) or siRNA control (sic). mRNA quantification was standardized with RNA 18S. Experiments were performed in biological triplicates and repeated three times. A representative experiment is shown (mean +- standard deviation). The knockdown efficiency, analyzed by Western blot, is shown in S2 File and was also checked by RT qPCR (not shown). (B, C) C2C12 cells co-transfected with bicistronic plasmids and 48h later with siRNA siM, sip54 or sic as above. *Renilla* luciferase (LucR, left) and firefly luciferase (LucF, right) mRNA levels were quantified by RT qPCR during C2C12 myoblast proliferation (grey histograms) and differentiation (black histograms), standardized to 18S RNA. The bicistronic cassette contains either the CMV promoter (B) or the FGF1 promoter A (C). For each experiment, values are shown relatively to the siRNA control. Experiments were performed in biological triplicates and repeated at least three times. A representative experiment is shown (mean +- standard deviation).

The role of p54^nrb^ and hnRNPM on FGF1 expression was studied by a knockdown approach using siRNA smartpools targeting either p54^nrb^ (sip54) or hnRNPM (siM) (S2 File). Myoblasts were co-transfected with bicistronic vectors containing the FGF1 IRES and siRNAs. The bicistronic cassette, coding for *renilla* luciferase (LucR) and firefly luciferase (LucF) separated by the FGF1 IRES, was under the control of either the CMV promoter or the FGF1 promoter A (Fig 3). Endogenous FGF1 mRNA was quantified by RT qPCR, and expression of the bicistronic mRNA under the control of either the CMV or FGF1 promoter was compared.

RT qPCR quantification of endogenous FGF1 mRNA clearly showed, as we have previously reported, a strong induction of FGF1 mRNA accumulation at day 2 of differentiation [4]. This induction was abolished upon transfection with siRNA against either hnRNPM or p54^nrb^ (Fig 3A and S2 File).

Bicistronic mRNA levels produced from the CMV or FGF1 promoter were quantified with LucF and LucR couples of primers. No variation of mRNA amount was observed with the CMV promoter in response to siRNA knockdown of hnRNPM or p54^nrb^ (Fig 3B). In contrast, the level of bicistronic mRNA transcribed from the FGF1 promoter was strongly downregulated by hnRNPM or p54^nrb^ knockdown in differentiating myoblasts (Fig 3C). In addition, the two cistrons LucR and LucF were present in equal amounts in all experiments, ruling out any effect of hnRNPM or p54^nrb^ on a putative cryptic splicing site or promoter in the IRES (Fig 3B and 3C).

These data showed, together with the ChIP data (Fig 2A), that hnRNPM and p54^nrb^/NONO binding to the FGF1 promoter A is responsible for FGF1 mRNA accumulation during myoblast differentiation, suggesting an effect of these proteins on the FGF1 mRNA transcription or stability.

### p54^nrb^ and hnRNPM knockdown silences the FGF1 IRES during myoblast differentiation

**Fig 4:**
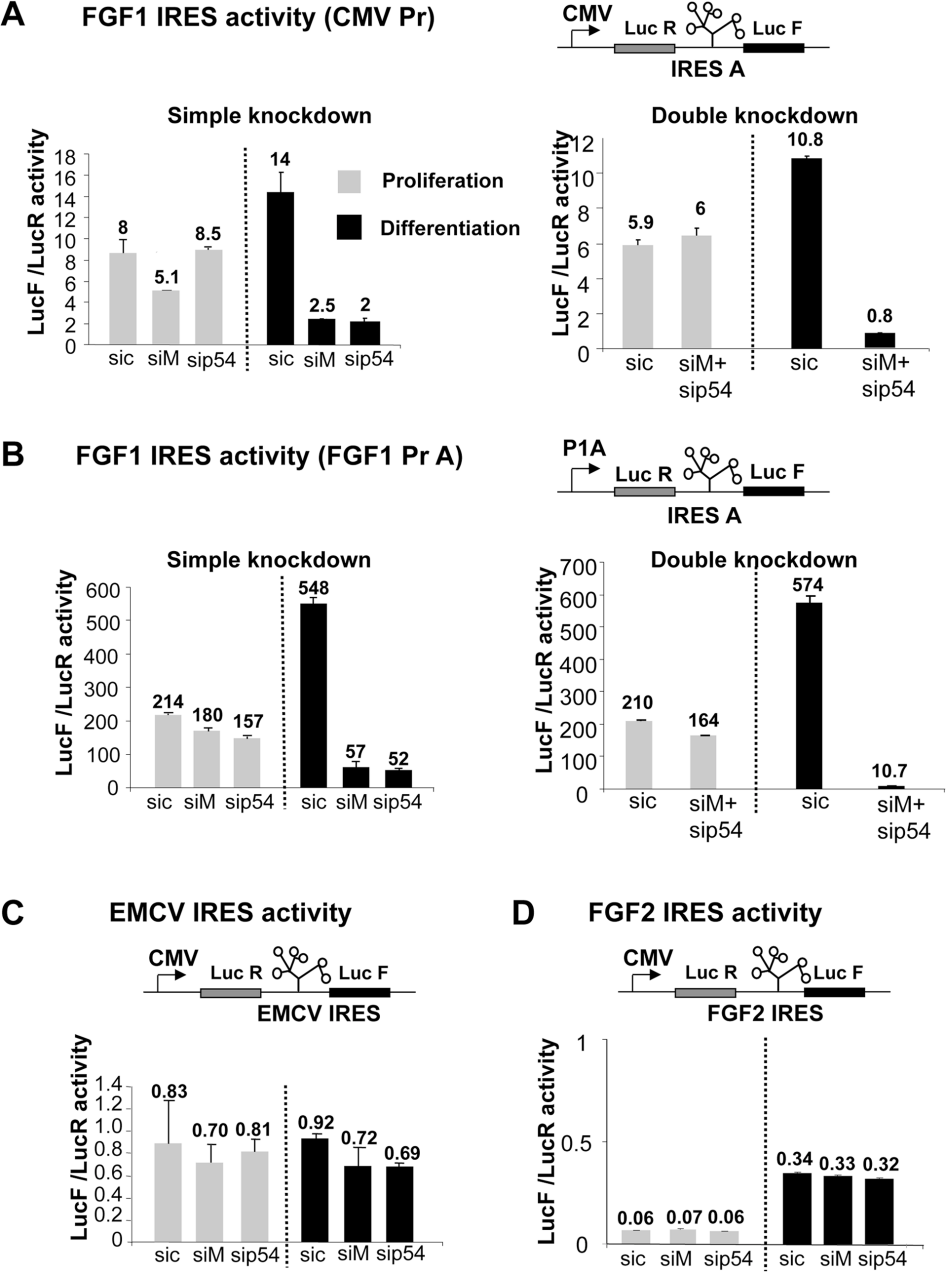
Effect of hnRNPM and/or p54^nrb^ knockdown on the regulation of FGF1 IRES A activity during myoblast differentiation. C2C12 cells were first transfected with bicistronic plasmids and 48h later with siRNAs targeting hnRNPM (siM), p54^nrb^ (sip54) or control (sic), (see Fig 3). Luciferase activities were measured as described in Mat. & Meth two days after siRNA transfection, from C2C12 myoblasts maintained in proliferation (grey histogram) or serum-starved to induce differentiation (day 2, black histogram). The bicistronic cassette contains LucR and LucF reporter genes, separated by different IRESs (FGF1, FGF2 or EMCV). LucR and LucF activities reflect the cap-dependent and IRES dependent translation, respectively [4]. The knockdown efficiency was checked by Western blot (S2 File). (A, B) FGF1 IRES activities were measured in C2C12 myoblasts in proliferation (grey histogram) and differentiation two days after serum-starvation treatment (black histogram). The bicistronic constructs are schematized. The bicistronic cassette is under the control of either the CMV promoter (A) or the FGF1 promoter A (B). Single and double knockdowns are shown in left and right panels, respectively. IRES activities are represented as LucF/LucR ratios. (C, D). Absence of effect of hnRNPM and/or p54^nrb^ knockdown on the regulation of EMCV and FGF2 IRESs during myoblast differentiation. Activities of EMCV (C) and FGF2 (D) IRESs were measured as above. For all panels, experiments were performed in biological triplicates and repeated at least three times. A representative experiment is shown (mean +- standard deviation).

The knockdown approach was also used to evaluate the effect of hnRNPM and p54^nrb^ on translation mediated by the FGF1 IRES A. In the bicistronic construct, LucR activity reflects the mRNA level and cap-dependent translation while LucF reflects the IRES activity. Luciferase activities were measured in myoblasts co-transfected with bicistronic vectors and siRNAs targeting hnRNPM and/or p54^nrb^ (see above, and S2 File). Under the CMV promoter, FGF1 IRES activity decreased by 5.6 or 7 fold following knockdown of hnRNPM or p54^nrb^, respectively (Fig 4A, left panel). A double knockdown resulted in a 13.5 fold inhibition (Fig 4A, right panel). These effects were observed in differentiating but not proliferating myoblasts. When the bicistronic cassette was under the control of the FGF1 promoter A, the IRES activity was 39 fold higher than with the CMV promoter (as published previously) and more strongly affected by hnRNPM or p54^nrb^ knockdown (8 and 11 fold, respectively, Fig 4B, left panel) [4]. In addition, the double knockdown drastically inhibited the IRES activity 54 fold (Fig 4B, right panel). Additional siRNAs targeting p54^nrb^ or hnRNPM, but not contained in the smartpools, were assessed, confirming that the effect of p54 or hnRNPM knockdown on FGF1 IRES activity is specific to these targets (S3 File).

To check the specificity of FGF1 IRES regulation by hnRNPM and p54^nrb^, knockdown was performed as above using bicistronic contructs with the EMCV or FGF2 IRES. Results showed that the activities of the EMCV and FGF2 IRESs remained completely unaffected by either knockdown (Fig 4C and 4D).

These data suggested that hnRNPM and p54^nrb^ participate in the IRESome complex responsible for FGF1 IRES A activation during myoblast differentiation, but are unable to activate EMCV or FGF2 IRESs. In addition, the presence of the FGF1 promoter A stimulated the IRES activating function of the two proteins, suggesting that the recruitment of hnRNPM and p54^nrb^ on the promoter by a direct or indirect interaction might enhance their recruitment in the IRESome.

### P54^nrb^ is sufficient to stimulate the FGF1 promoter whereas it cooperates with hnRNPM to activate the FGF1 IRES

**Fig 5:**
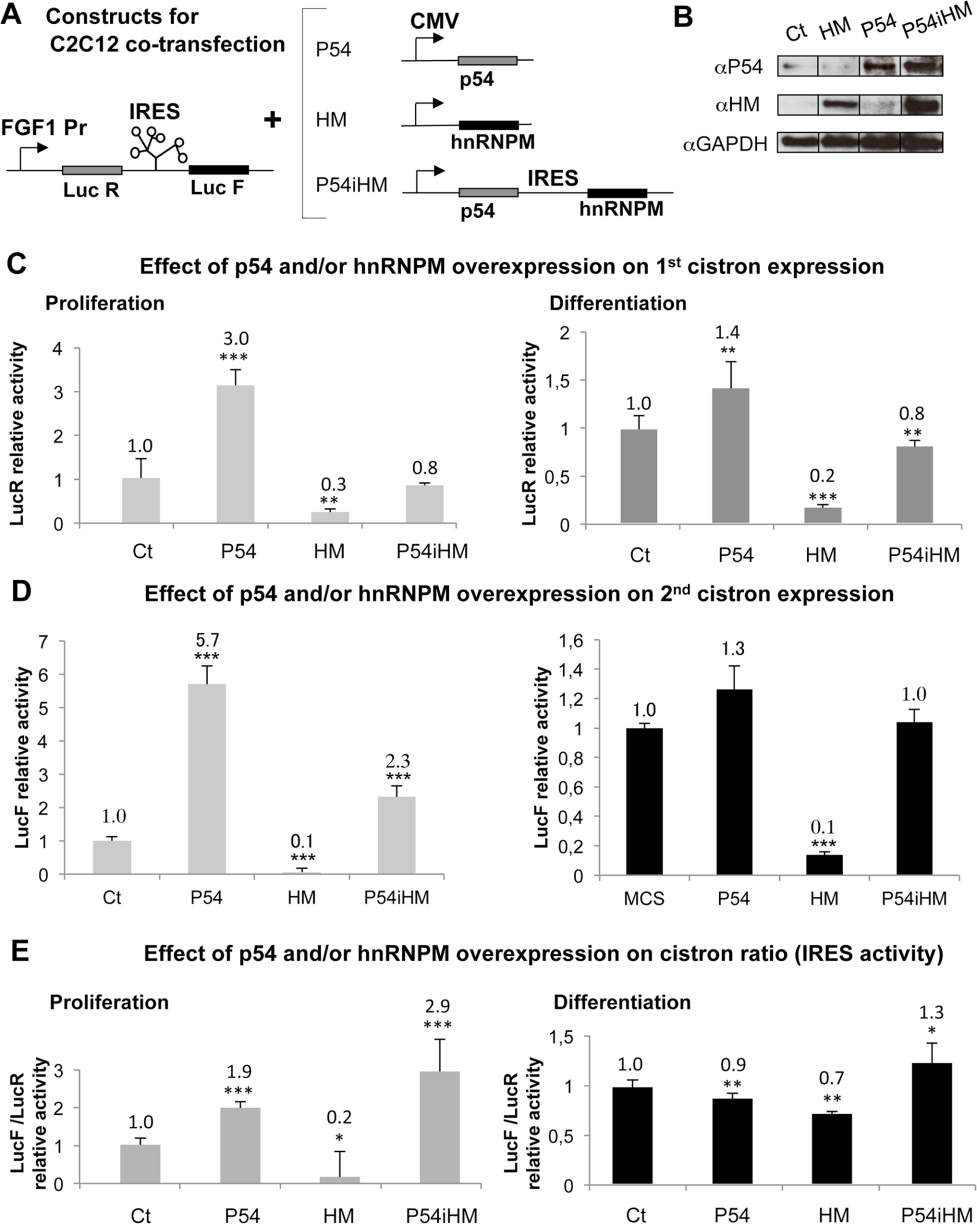
Cooperative effect of p54^nrb^ and hnRNPM on FGF1 IRES activation. (A) Schema of the constructs used for C2C12 cell co-transfection. The target plasmid is the bicistronic dual luciferase vector with the FGF1 promoter and IRES. The effector plasmids express p54^nrb^ (p54) or hnRNPM (HM), or co-express p54^nrb^ and hnRNPM. The latter plasmid is a bicistronic contruct containing the FGF1 IRES. (B) Western blots of transfected proliferating C2C12 cell extracts using antibodies against p54(αHM), hnRNPM (αHM) of GAPDH as a control (αGAPDH). (C-E) Luciferase activities measurement of co-transfected proliferating or differentiating C2C12 cell extracts. LucR activity reflects the FGF1 mRNA promoter activity (as cap-dependent translation does not significantly vary, as shown by RNA transfection, see Table B in S4 File) (C). LucF reflects IRES-dependent translation but is also dependent on mRNA amount (D). LucF/LucR ratio reflects the IRES activity normalized to mRNA amount, expressed relatively to the control (co-transfection with empty vector) (E). Experiments were performed in biological triplicates and repeated three times. The statistical test used is the Student test. (mean +- standard deviation, *p<0.05, **p<0.01, ***<0.001).

To evaluate the double activity of p54^nrb^ and hnRNPM on FGF1 promoter and IRES, C2C12 myoblasts were co-transfected by the bicistronic FGF1 IRES-containing dual luciferase plasmid and by plasmids coding either p54^nrb^ or hnRNPM, or both proteins (Fig 5A and 5B). Measurement of the LucR activity showed significant mRNA accumulation in response to p54^nrb^ overexpression (Fig 5C). The IRES activity reflected by the ratio LucF/LucR was enhanced by p54^nrb^ alone, but more strongly with the combination of p54^nrb^ and hnRNPM (Fig 5D and 5E). Surprisingly, overexpression of hnRNPM alone inhibited both mRNA accumulation and IRES activities, suggesting that the ratio of the two proteins may be important for hnRNPM function (Fig 5C–5E). The effects of protein overexpression were stronger in proliferating myoblasts, which could be expected, because promoter and IRES are in a basal inactive state in proliferating cells, while they are activated by the endogenous p54^nrb^ and hnRNPM in differentiating cells. These results suggested that p54^nrb^ is sufficient to enhance mRNA accumulation, whereas it cooperates with hnRNPM to stimulate the IRES. Such a cooperative effect confirmed the data of the knockdown experiments (Figs 3 and 4).

### Regulation of IRES activity by hnRNPM and p54^nrb^ is promoter-dependent

**Fig 6:**
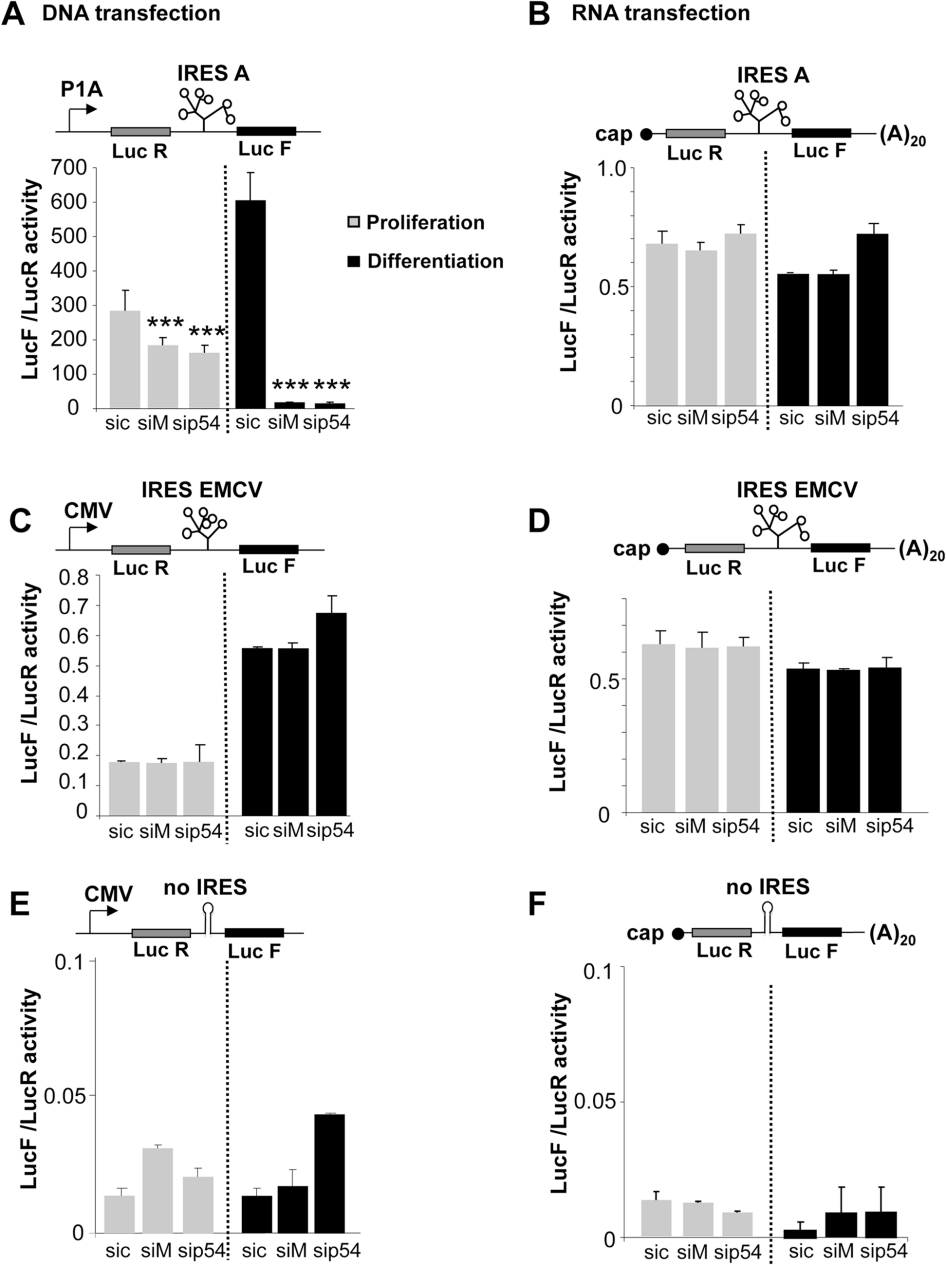
Comparison of IRES activities following DNA or RNA transfection. (A, C, E) C2C12 cells were transfected with bicistronic plasmids containing either the FGF1 IRES, or EMCV IRES or a hairpin (control without IRES) and with siRNA siM, sip54 or sic, and IRES activities measured as in Fig 4B, 4D and 4F C2C12 cells were transfected with siRNA siM, sip54 or sic and 24h later with bicistronic mRNAs containing either the FGF1 IRES, or the EMCV IRES, or a hairpin as above. mRNAs were transcribed in vitro, capped and polyadenylated, as described in Mat. & Meth. The LucF/LucR activity ratio was measured 12h post-transfection. Experiments were performed in biological triplicates and repeated three times. The Student test was used. A representative experiment is shown (mean +- standard deviation, *p<0.05, **p<0.01, ***<0.001). For A and B, the Luc R and Luc F values are shown in S4 File.

To analyse the impact of the promoter on activation of IRES-dependent translation by hnRNPM and p54^nrb^, C2C12 cells were transfected with either bicistronic plasmid DNAs or in vitro transcribed capped and polyadenylated bicistronic mRNAs (Fig 6). DNA transfection resulted (as already shown in Fig 4) in a high FGF1 IRES activity, strongly induced by C2C12 differentiation, and downregulated by the knockdown of hnRNPM or p54^nrb^ (Fig 6A and S4 File). In contrast, basal FGF1 IRES activity was 500 times lower following RNA transfection, and no induction by either hnRNPM or p54 was observed (Fig 6B and S4 File). These features were not observed with the EMCV IRES, whose activity was low after DNA or RNA transfection and was not affected by hnRNPM or p54 knockdown (Fig 6C and 6D). As a negative control, we used a construct containing a hairpin between the two cistrons instead of an IRES. The LucF/LucR ratios in the absence of an IRES were very low for both DNA and RNA transfections and were not affected by hnRNPM or p54 knockdown (Fig 6E and 6F). These results suggested that a nuclear step may be a prerequisite to IRES induction by the hnRNPM and p54^nrb^.

**Fig 7:**
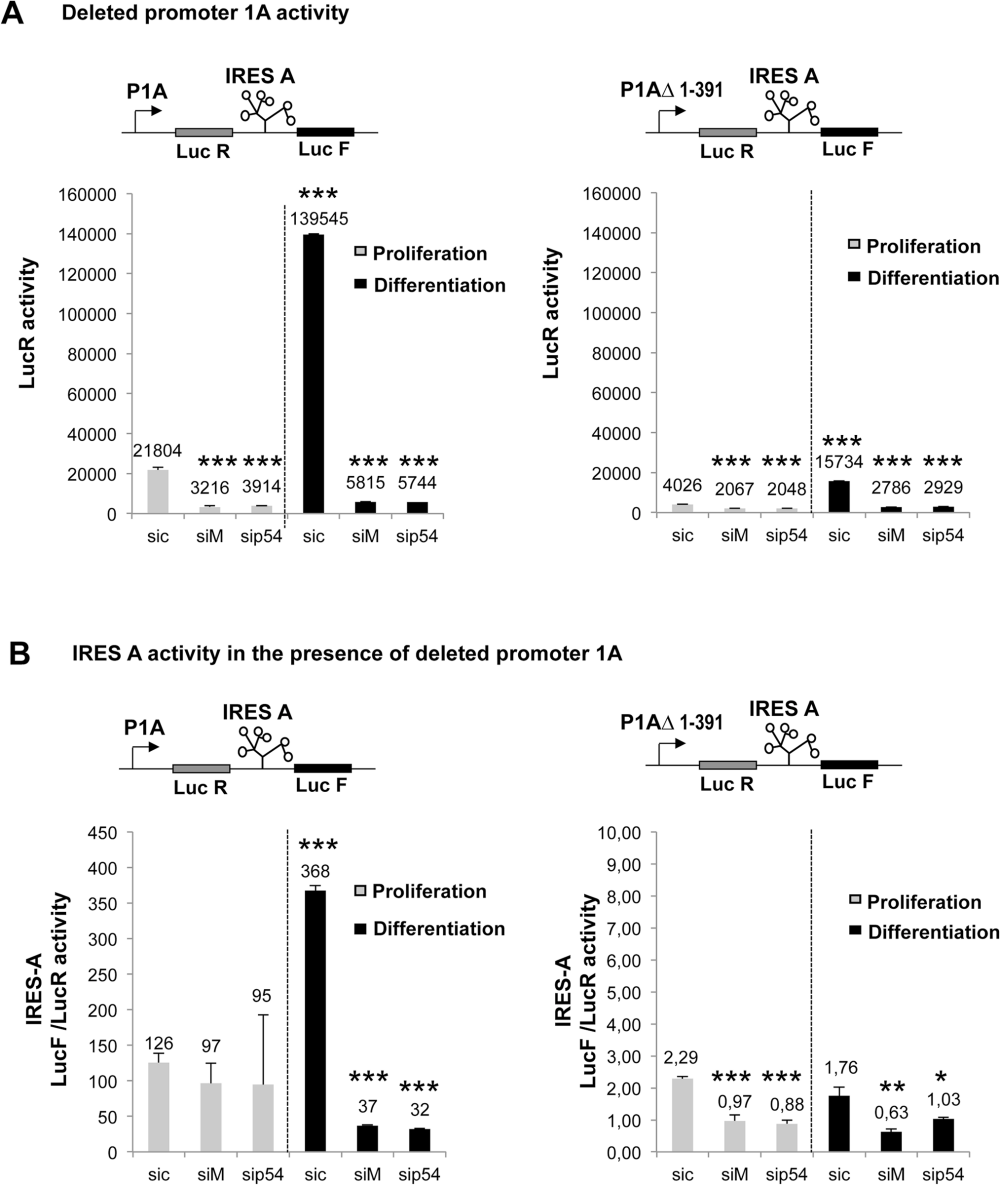
Influence of transcription level on the activity of the FGF1 IRES A. C2C12 cells were transfected with bicistronic plasmids containing either the complete promoter 1A or a deleted promoter lacking nucleotides 1 to 391. Transfected cells were treated 24h later with siRNA siM, sip54 or sic, and luciferase activities were measured as in Fig 6. (A) mRNA expression in the presence of promoter 1A and 1AΔ1–391 reflected by the LucR activities. (B) Activity of FGF1 IRES A in the presence of promoter 1A and 1AΔ1–391, reflected by the Luc F/ LucR ratio as above. Experiments were performed in biological triplicates and repeated three times. The statistical test used is the Student test. (mean +- standard deviation, *p<0.05, **p<0.01, ***<0.001).

To go further with this hypothesis, we transfected C2C12 with a construct containing a deleted form of promoter F1A, P1AΔ1–391 (Fig 7). Measurements of LucR activity showed that this promoter is about ten times less efficient than the wild type promoter, whereas it was still sensitive to hnRNPM and p54 knockdown (Fig 7A). In contrast, in the presence of the deleted form of the promoter, IRES activity was no longer induced and was very mildly altered by the knockdown of hnRNPM or p54^nrb^ (Fig 7B).

These two sets of data showed that FGF1 IRES induction by hnRNPM and p54^nrb^ depends on a sequence present in the promoter and involves multifunctional roles of these proteins.

### p54^nrb^ and hnRNPM are required for FGF1 induction and myotube formation

**Fig 8:**
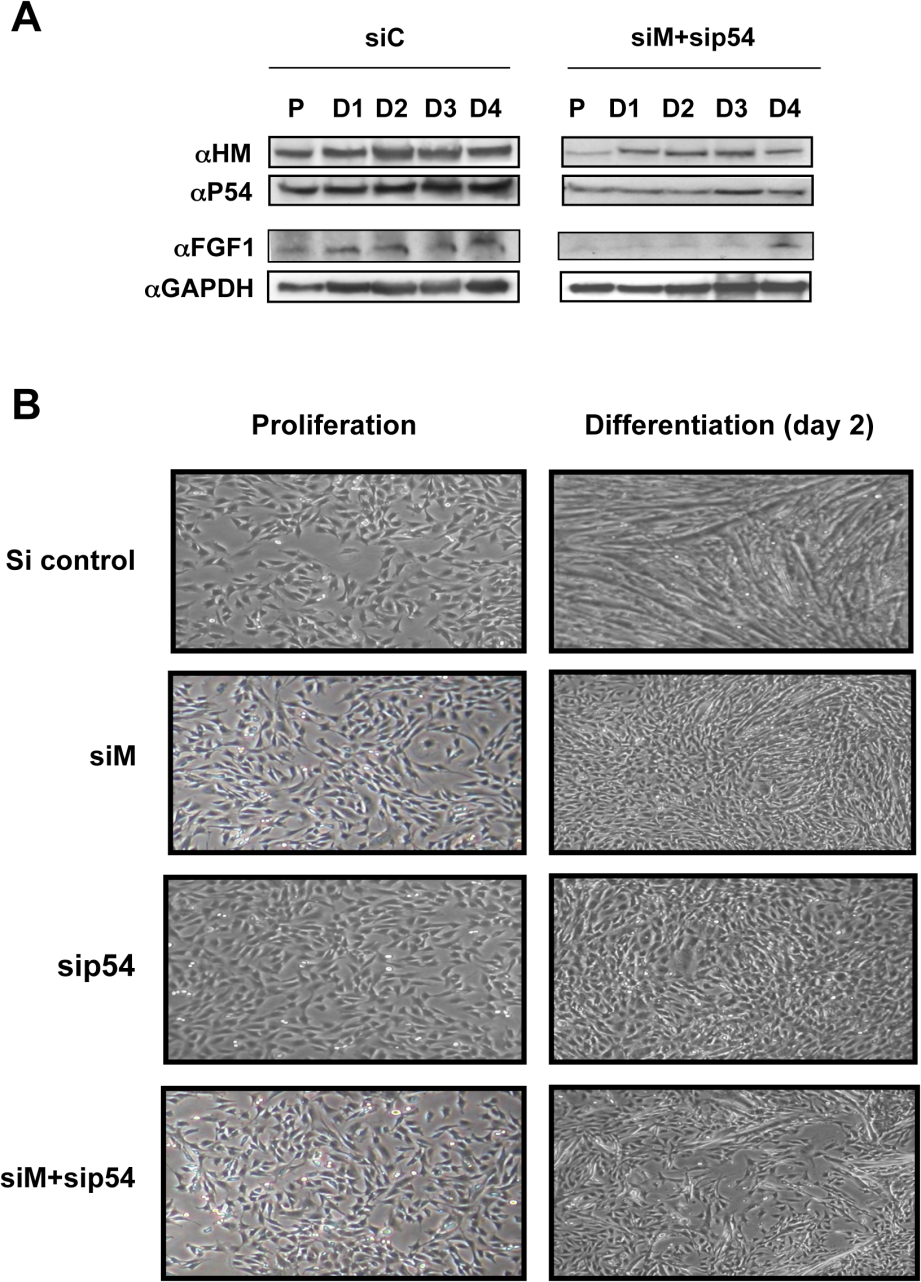
Role of hnRNPM and p54^nrb^ in myoblast differentiation. (A) Effect of hnRNPM and p54^nrb^ knockdown on expression of endogenous FGF1. C2C12 cells were transfected with siRNA, siM+sip54 or sic. Western blot was performed as above using cell extracts of proliferating (P) and differentiating myoblasts (D1 to D4). GAPDH was used as a normalization control. These data correspond to a representative experiment (repeated at least three times). (B) Effect of hnRNPM and/or p54^nrb^ knockdown on myotube formation. C2C12 cells were transfected with siRNA siM, sip54, siM+sip54 or sic and myotube formation was followed by phase contrast microscopy. Data are shown for day 2 cells compared to proliferative cells.

To determine whether p54^nrb^ and hnRNPM are responsible for endogenous FGF1 induction during differentiation and if these proteins exhibit a physiological role in the development of muscle fiber, simple and double knockdowns of hnRNPM and/or p54^nrb^ were performed (Fig 8A). Expression of FGF1, analyzed by Western Blot, was strongly inhibited by the simple or double knockdown (Fig 8A and data not shown). Furthermore, formation of myotubes was drastically affected by knockdown of p54^nrb^ or hnRNPM, and even more strongly inhibited by the double knockdown (Fig 8B). Decrease of endogenous FGF1 expression upon treatment with siRNA against p54^nrb^ and/or hnRNPM confirmed that these two proteins are involved in the control of FGF1 expression and consequently in its induction during differentiation. In addition, these data also revealed that p54^nrb^ and hnRNPM are required for differentiation of myoblasts into myotubes, revealing the important role of these proteins in myogenesis.

## Discussion

The present data reveal that FGF1 IRES activity is promoter-dependent and that this process is governed by two multifunctional trans-acting factors, p54^nrb^ and hnRNPM. P54^nrb^ and hnRNPM also appear as crucial actors of myoblast differentiation, demonstrating the physiological relevance of this new mechanism.

### Nuclear formation of the IRESome

The present data suggest that IRESome formation occurs in the nuclear compartment. The regulating features of p54^nrb^ and hnRNPM on both promoter and IRES activities are not independent events, as these two translational regulators do not work when the promoter is absent (RNA transfection) or inactivated (promoter mutation).

The importance of ITAF subcellular localization in the regulation of their activity appeared a few years ago, with the first observation that a nuclear event is required in the control of c-myc IRES activity [18]. Later, it has been shown that shuttling of ITAFs, such as hnRNP A1, RBM4, PTB and PCBP1, between the nucleus and the cytoplasm influences their activity [24, 27, 30, 41]. Two different mechanisms have been proposed [42]: 1) the nuclear localized ITAFs may associate with their target in this compartment, resulting in the sequestration of the mRNA in the nucleus until an appropriate signal occurs, allowing cytoplasmic export and translation, 2) the ITAFs may be primarily located in the nucleus to keep them separate from their target IRESs and await a signal that warrants their accumulation in the cytoplasm. These two models could apply to cellular and viral IRESs, respectively, as explained by Semler & Waterman who argue that nuclear versus cytoplasmic synthesis of IRES-containing mRNAs is a major determinant in assembling different RNA–protein complexes [43]. The synthesis of uncapped picornaviral mRNAs occurs in the cytoplasm, in contrast to cellular mRNAs, which are transcribed in the nucleus and then exported to the cytoplasm. Our study not only reinforces the hypothesis that the IRESome is constituted in the nucleus, but further elaborates this model by showing that its formation is influenced by the promoter activity.

### HnRNPM and p54^nrb^ cooperate in activation of IRES-dependent translation

The IRES-regulating function of hnRNPM discovered in our study represents a novel finding, as well as its cooperative activity with p54^nrb^. Interestingly, nuclear interaction of hnRNPM and p54^nrb^ has been previously reported [40]. Like other hnRNPs, hnRNPM has been first identified as a splicing regulatory protein, associated with early spliceosomes where it modulates the alternative splicing pattern of specific mRNAs including the FGF receptor [39, 40]. Interestingly, hnRNPM has also been reported to influence the localization of *nanos* mRNA by binding to its 3' UTR [44]. The additional function of hnRNPM shown in our study confirms that hnRNPs are multifunctional, depending on their interacting partners.

p54^nrb^/NONO is also a multifunctional protein. It has been previously shown to associate with 5' splicing sites in large transcription-splicing complexes, providing a direct role for this protein in linking transcription to splicing [3, 45]. P54^nrb^ also behaves as a specific transcriptional activator, binding to the intracisternal A particle proximal enhancer element, and as an RNA binding protein, preferentially binding to the sequence UAGGGA/U identified by SELEX [37]. In addition, p54^nrb^ has been identified as an ITAF of the myc family IRESs (c-, L- and N-myc), and has also been shown to stimulate the APAF-1 IRES [46]. Here we demonstrate that p54^nrb^ is able to cooperate with hnRNPM, suggesting that the two proteins may activate the FGF1 IRES as a complex. Unexpectedly, hnRNPM overexpression is inhibitory, whereas knockdown experiments clearly showed it is required for both IRES and promoter activation. We hypothesize that excess of hnRNPM might inhibit p54^nrb^ functions on FGF1 gene expression, presumably by preventing p54^nrb^ binding to FGF1 DNA and RNA due to an inadequate stoechiometry of the two proteins.

Although the proteins are expressed in proliferating cells, they are not found in the complex with the IRES or the promoter A. This suggests that the proteins might carry different post-translational modifications. Alternatively these proteins could join the complex through the interaction with another protein, which is absent in the proliferating cells and remains to be discovered. This could also explain that overexpressed hnRNPM is not functional.

Very few proteins were bound to the EMCV IRES, which may be explained by the low efficiency of the EMCV IRES in myoblasts. However, we were also surprised that no transcription factor was bound to promoter DNA. This indicates that BIA-MS does not provide an exhaustive view of all proteins bound to RNA or DNA and that one can miss important proteins. This also explains that we have missed hnRNPM present in the promoter-bound complex.

Although the direct interaction of p54^nrb^ and hnRNPM with RNA has not been demonstrated, the present study strongly suggest that the two proteins are ITAFs involved in FGF1 IRES activation. However The BIA-MS data provides a list of additional proteins bound to the FGF1 IRES, including histones and splicing factors. These proteins may participate in the IRESome ribonucleoprotein.

### Promoter-dependent activation of the FGF1 IRES may involve transcription or mRNA stabilization

Promoter-dependent translation controlled by hnRNPM and p54^nrb^ asks the question of how a promoter is able to impact on translation, which is in principle cytoplasmic.

One among several possible models, one can propose transcription/translation coupling: hnRNPM and p54^nrb^ might be first recruited to the promoter, where they would contribute to transcription activation. This process would also facilitate hnRNPM and p54^nrb^ binding onto mRNA IRES and subsequently activate IRES-dependent translation. A recent study supports the hypothesis of a co-transcriptional ITAF binding, by showing that the translation elongation factor eEF1A1 couples transcription to translation during heat shock response [47]. These authors have demonstrated that eEF1A1 activates HSP70 transcription, then associates with RNA polymerase II, binds the mRNA 3’UTR, stabilizes it and facilitates its nuclear export to active ribosomes. hnRNPM and p54^nrb^ may well have a similar mode of action, except that they bind to the mRNA 5’UTR and directly activate IRES-dependent translation by their ITAF function.

Other hnRNPs have been previously described for their involvement in transcription: hnRNPK, reported as a transcription factor interacting with the RNA polymerase II machinery in the c-myc promoter, and hnRNP A1, involved in the transcription of the KRAS proto-oncogene [48, 49]. More recently, hnRNPC has been shown to regulate transcription in osteoblasts [50]. Interestingly, hnRNP A1 and-K also exhibit an ITAF function, although no coupling between transcriptional and translational functions has been mentioned [24, 25, 30].

An alternative model to explain promoter-dependent regulation of translation by hnRNPM and p54^nrb^ might be their effect on mRNA stability. Binding of these proteins to the FGF1 promoter would not have an effect on transcription but would facilitate their binding to the nascent mRNA, resulting in mRNA stabilization, as has been shown for the yeast protein Dbf2P [51]. These authors demonstrate that Dbf2p is recruited to the SW15 and CBL2 promoters, and co-transcriptionally deposited onto the SW15 and CLB2 mRNAs. Once they are exported into the cytoplasm, the two mRNAs are protected from degradation by Dbf2p. According to this model, hnRNPM and p54^nrb^ binding to mRNA might have the double effect of stabilizing the FGF1 mRNA and activating the IRES. Anyway, further investigation is needed to determine whether the promoter-dependent function of hnRNPM and p54^nrb^ involves transcription enhancement or RNA stabilization.

### Promoter-dependent translation, a physiologically relevant mechanism during myogenesis

An important feature of our study is the physiological relevance of p54^nrb^ and hnRNPM function in myoblast differentiation. It clearly appears that p54^nrb^ and hnRNPM are important for myotube formation and such a role is novel for these two proteins. We have previously shown that FGF1 is required for myoblast differentiation [4]. Thus the effect of hnRNPM and/or p54 knockdown on myoblast differentiation may be a direct consequence of FGF1 downregulation. Alternatively, hnRNPM and p54^nrb^ may act on the expression of additional IRES-containing genes, including Smad5 and utrophin A IRESs whose activites have been reported in myoblasts [17, 52, 53]. It will be of great interest to investigate the possible role of hnRNPM and p54^nrb^ in the promoter-dependent regulation of such IRESs during muscle development.

As seen with the coordination of transcription and mRNA processing, such as splicing, whose regulators often exhibit the additional function of ITAFs (i.e. hnRNPs), one can expect that the promoter-dependent translation mechanism described in our study will extend to other IRES-containing mRNAs. Such a mechanism becomes more significant for genes that are induced in response to conditions that block cap-dependent translation, such as stress. Coupling of IRES activation with the promoter guarantees that the induction of a given gene in response to a specific stimulus will result in the synthesis of that gene product.

## Supporting Information

S1 File Identification of RNA and DNA binding proteins by BIA-MS. Total or nuclear extracts of differentiating C2C12 myoblasts were injected (see Fig 1) as analytes in several BIACORE 3000 channels after immobilization of ligands corresponding to FGF1 IRES A, EMCV IRES, FGF1 promoter A or CMV promoter. Bound proteins were recovered as described in Mat. & Meth. and identified by mass spectrometry. For each BIA-MS experiment, 6 recovery cycles were pooled to obtain a sufficient RU quantity (about 2000 RU). Mass spectrometry analysis was performed as described in Mat. & Meth. Bound proteins (RNA and DNA binding proteins) are listed here. (Table A) FGF1 IRES RNA, C2C12 total extracts, (Table B) FGF1 IRES RNA, C2C12 nuclear extracts, (Table C) EMCV IRES RNA, C2C12 total extracts, (Table D) FGF1 promoter A DNA, C2C12 nuclearFGF1 extracts.
(DOC)

S2 File Knockdown of hnRNPM and/or p54^nrb^. C2C12 cells were first transfected with bicistronic plasmids and 48h later with siRNAs targeting hnRNPM (siM), p54^nrb^ (sip54) or control (sic). RNA levels and luciferase activities are presented in Figs 3 and 4, respectively, from C2C12 myoblasts maintained in proliferation or at day 2 of differentiation. p54^nrb^ and hnRNPM expression was analysed by Western blot following single knockdown with siRNA siM (Fig A) or sip54 (Fig B) or double knockdown with the two siRNAs together (Fig C).
(TIF)

S3 File Effect of hnRNPM and/or p54^nrb^ knockdown on the regulation of FGF1 IRES A using siRNAs different from the smartpool. C2C12 cells co-transfected with bicistronic plasmids and 48h later with a siRNA against hnRNPM (siM), p54^nrb^ (sip54) or siRNA control (sic). SiM and siP54 corresponded to sequences different from that of the siRNA smartpools used in Fig 4. (Fig A) Ratio of the luciferase activities measured as in Fig 4, two days after siRNA transfection, from differentiating C2C12 myoblasts (day 2). (Fig B) The knockdown was checked by Western blot as in S2 File.
(TIF)

S4 File Effect of hnRNPM and p54 knockdown on FGF1 IRES activity after DNA or RNA transfection. (Table A) C2C12 cells were transfected with bicistronic plasmids containing the FGF1 promoter and IRES (see Fig 6) and with siRNA siM, sip54 or sic. (Table B) C2C12 cells were transfected with siRNA siM, sip54 or sic and 24h later with bicistronic mRNA containing the FGF1 IRES (see Fig 6). mRNAs were transcribed in vitro, capped and polyadenylated, as described in Mat. & Meth. For Tables A and B, firefly and *renilla* luciferase activities were measured. Values are presented as well as the LucF/LucR (F/R) ratio representing the IRES activity. Experiments were performed in biological triplicates and repeated three times. The Student test was used (mean +- standard deviation).
(DOC)
