# Supplementary material for: Promoter-Dependent Translation Controlled by p54nrb and hnRNPM during Myoblast Differentiation
Source: PLoS One. 2015 Sep 2;10(9):e0136466. doi: 10.1371/journal.pone.0136466 (PMC4558007; doi:10.1371/journal.pone.0136466)
Supplement: S1 File — Total or nuclear extracts of differentiating C2C12 myoblasts were injected (see Fig 1) as analytes in several BIACORE 3000 channels after immobilization of ligands corresponding to FGF1 IRES A, EMCV IRES, FGF1 promoter A or CMV promoter. Bound proteins were recovered as described in Mat. & Meth. and identified by mass spectrometry. For each BIA-MS experiment, 6 recovery cycles were pooled to obtain a sufficient RU quantity (about 2000 RU). Mass spectrometry analysis was performed as described in Mat. & Meth. Bound proteins (RNA and DNA binding proteins) are listed here. (Table A) FGF1 IRES RNA, C2C12 total extracts, (Table B) FGF1 IRES RNA, C2C12 nuclear extracts, (Table C) EMCV IRES RNA, C2C12 total extracts, (Table D) FGF1 promoter A DNA, C2C12 nuclearFGF1 extracts. (DOC) [file pone.0136466.s001.doc]

Ainaoui et al, S1 file.

A: BIA-MS data: total C2C12 extract proteins bound to the FGF1 IRES A RNA.

| AC | ID | Description | MW | pI | Score | Peptides | Protein Coverage (%) |
| --- | --- | --- | --- | --- | --- | --- | --- |
| P20152 | VIME_MOUSE | Vimentin | 53655.06 | 5.06 | 400.67 | 27 | 52.4 |
| P62737 | ACTA_MOUSE | Actin. aortic smooth muscle | 41981.81 | 5.23 | 270.48 | 15 | 10.1 |
| P60710 | ACTB_MOUSE | Actin. cytoplasmic 1 | 41709.73 | 5.29 | 258.1 | 15 | 10.2 |
| P99024 | TBB5_MOUSE | Tubulin beta-5 chain | 49638.97 | 4.78 | 120.76 | 5 | 2.9 |
| P62908 | RS3_MOUSE | 40S ribosomal protein S3 | 26657.41 | 9.68 | 112.42 | 6 | 11.4 |
| P62264 | RS14_MOUSE | 40S ribosomal protein S14 | 16262.53 | 10.07 | 88.8 | 2 | 10.5 |
| P31001 | DESM_MOUSE | Desmin | 53465.04 | 5.21 | 87.77 | 5 | 1.8 |
| P43277 | H13_MOUSE | Histone H1.3 | 22086.13 | 11.03 | 85.81 | 6 | 4.7 |
| P43274 | H14_MOUSE | Histone H1.4 | 21964.02 | 11.1 | 84.73 | 5 | 21.9 |
| Q8BFZ3 | ACTBL_MOUSE | Beta-actin-like protein 2 | 41976.99 | 5.3 | 76.41 | 8 | 20.5 |
| Q9D0E1 | HNRPM_MOUSE | Heterogeneous nuclear ribonucleoprotein M | 77597.38 | 8.8 | 72.2 | 15 | 17 |
| P10126 | EF1A1_MOUSE | Elongation factor 1-alpha 1 | 50082.1 | 9.1 | 72.14 | 6 | 2.4 |
| P62751 | RL23A_MOUSE | 60S ribosomal protein L23a | 17684.13 | 10.44 | 70.52 | 1 | 5.3 |
| P99027 | RLA2_MOUSE | 60S acidic ribosomal protein P2 | 11643.83 | 4.42 | 69.92 | 5 | 60 |
| P62852 | RS25_MOUSE | 40S ribosomal protein S25 | 13733.7 | 10.12 | 65.11 | 4 | 22.4 |
| P15331 | PERI_MOUSE | Peripherin | 54234.59 | 5.4 | 63.83 | 3 | 1.1 |
| Q922F4 | TBB6_MOUSE | Tubulin beta-6 chain | 50058.13 | 4.8 | 60.29 | 3 | 6.7 |
| P10107 | ANXA1_MOUSE | Annexin A1 | 38709.97 | 6.97 | 57.9 | 3 | 10.7 |
| P16858 | G3P_MOUSE | Glyceraldehyde-3-phosphate dehydrogenase | 35787.21 | 8.44 | 57.42 | 9 | 22.5 |
| P62918 | RL8_MOUSE | 60S ribosomal protein L8 | 28007.29 | 11.03 | 56.28 | 3 | 5.1 |
| P17182 | ENOA_MOUSE | Alpha-enolase | 47111.21 | 6.37 | 53.17 | 6 | 11.5 |
| Q07133 | H1T_MOUSE | Histone H1t | 21527.15 | 11.71 | 52.28 | 3 | 13.5 |
| P47963 | RL13_MOUSE | 60S ribosomal protein L13 | 24290.5 | 11.54 | 49.61 | 2 | 10.9 |
| P61255 | RL26_MOUSE | 60S ribosomal protein L26 | 17247.53 | 10.55 | 42.74 | 2 | 4.9 |
| P11499 | HS90B_MOUSE | Heat shock protein HSP 90-beta | 83273.12 | 4.97 | 42.71 | 3 | 4.8 |
| P43276 | H15_MOUSE | Histone H1.5 | 22562.44 | 10.91 | 42.06 | 4 | 7.8 |
| Q61937 | NPM_MOUSE | Nucleophosmin | 32539.81 | 4.62 | 41.88 | 6 | 18.2 |
| Q9CQN1 | TRAP1_MOUSE | Heat shock protein 75 kDa, mitochondrial | 80158.53 | 6.25 | 41.74 | 1 | 0.3 |
| Q64475 | H2B1B_MOUSE | Histone H2B type 1-B | 13943.56 | 10.31 | 40.93 | 3 | 23 |
| P61358 | RL27_MOUSE | 60S ribosomal protein L27 | 15787.75 | 10.56 | 37.85 | 2 | 9.2 |
| P62900 | RL31_MOUSE | 60S ribosomal protein L31 | 14453.93 | 10.54 | 36.02 | 3 | 12.2 |
| P62806 | H4_MOUSE | Histone H4 | 11360.38 | 11.36 | 35.55 | 2 | 18.8 |
| P35700 | PRDX1_MOUSE | Peroxiredoxin-1 | 22162.35 | 8.26 | 31.21 | 3 | 15.1 |
| O08807 | PRDX4_MOUSE | Peroxiredoxin-4 | 31033.14 | 6.67 | 31.21 | 2 | 2.6 |
| P68369 | TBA1A_MOUSE | Tubulin alpha-1A chain | 50103.61 | 4.94 | 25.46 | 5 | 2.5 |

B: BIA-MS data: nuclear C2C12 extract proteins bound to the FGF1 IRES A RNA.

| AC | ID | Description | MW | pI | Score | Peptides | Protein Coverage (%) |
| --- | --- | --- | --- | --- | --- | --- | --- |
| P60710 | ACTB_MOUSE | Actin, cytoplasmic 1 | 41709.73 | 5.29 | 1487.66 | 20 | 15.2 |
| P68033 | ACTC_MOUSE | Actin, alpha cardiac muscle 1 | 41991.88 | 5.23 | 1216.7 | 19 | 14.8 |
| Q62093 | SFRS2_MOUSE | Splicing factor. arginine/serine-rich 2 | 25461.18 | 11.86 | 709.72 | 7 | 13.1 |
| Q8BFZ3 | ACTBL_MOUSE | Beta-actin-like protein 2 | 41976.99 | 5.3 | 591.13 | 11 | 30.6 |
| P56480 | ATPB_MOUSE | ATP synthase subunit beta, mitochondrial | 56265.47 | 5.19 | 561.29 | 16 | 47.1 |
| Q99M28 | RNPS1_MOUSE | RNA-binding protein with serine-rich domain 1 | 34187.7 | 11.85 | 337.96 | 5 | 7.6 |
| Q03265 | ATPA_MOUSE | ATP synthase subunit alpha. mitochondrial | 59715.59 | 9.22 | 230.42 | 9 | 21.7 |
| P63038 | CH60_MOUSE | 60 kDa heat shock protein, mitochondrial | 60917.39 | 5.91 | 221.49 | 6 | 20.1 |
| P24369 | PPIB_MOUSE | Peptidyl-prolyl cis-trans isomerase B | 23698.56 | 9.56 | 209.89 | 10 | 44.4 |
| Q569Z6 | TR150_MOUSE | Thyroid hormone receptor-associated protein 3 | 108113.69 | 10.17 | 142.45 | 8 | 10.8 |
| P19324 | SERPH_MOUSE | Serpin H1 | 46560.2 | 8.9 | 111.84 | 5 | 17.5 |
| Q52KI8 | SRRM1_MOUSE | Serine/arginine repetitive matrix protein 1 | 106828.46 | 11.87 | 86.78 | 6 | 7.9 |
| P62315 | SMD1_MOUSE | Small nuclear ribonucleoprotein Sm D1 | 13273.36 | 11.56 | 84.79 | 1 | 14.1 |
| Q8BG05 | ROA3_MOUSE | Heterogeneous nuclear ribonucleoprotein A3 | 39627.61 | 9.1 | 72.53 | 4 | 3.4 |
| Q6PDM2 | SFRS1_MOUSE | Splicing factor, arginine/serine-rich 1 | 27727.81 | 10.37 | 71.53 | 9 | 16.6 |
| P26369 | U2AF2_MOUSE | Splicing factor U2AF 65 kDa subunit | 53483.22 | 9.19 | 60.31 | 1 | 1.1 |
| Q99K48 | NONO_MOUSE | p54^nrb^/NONO Non-POU domain-containing octamer-binding protein | 54506.42 | 9.01 | 56.11 | 2 | 1.3 |
| P67984 | RL22_MOUSE | 60S ribosomal protein L22 | 14749.77 | 9.21 | 55.76 | 2 | 17.1 |
| O08810 | U5S1_MOUSE | 116 kDa U5 small nuclear ribonucleoprotein component EFTUD2 | 109291.3 | 4.86 | 43.05 | 1 | 0.2 |

C: BIA-MS data: total C2C12 extract proteins bound to the EMCV IRES RNA.

| AC | ID | Description | MW | pI | Score | Peptides | Protein Coverage (%) |
| --- | --- | --- | --- | --- | --- | --- | --- |
| P645534 | ACTA2_MOUSE | Actin alpha 2 smooth muscle | 16748,18 | 5,31 | 76,71 | 2 | 13,9 |
| P8603 | ACTA-MOUSE | Actin alpha smooth muscle | 41981,81 | 5,23 | 76,33 | 3 | 7,4 |
| P27547 | DCD_MOUSE | dermcidin | 11276,83 | 6,08 | 60,65 | 1 | 10 |

D: BIA-MS data: nuclear C2C12 extract proteins bound to the FGF1 promoter A DNA.

| AC | ID | Description | MW | pI | Score | Peptides | Protein Coverage (%) |
| --- | --- | --- | --- | --- | --- | --- | --- |
| P03995 | GFAP_MOUSE | Glial fibrillary acidic protein | 49869.5 | 5.27 | 995.36 | 5 | 9.5 |
| P60710 | ACTB_MOUSE | Actin. cytoplasmic 1 | 41709.7 | 5.29 | 247.21 | 9 | 6.9 |
| P63038 | CH60_MOUSE | 60 kDa heat shock protein, mitochondrial | 60917.4 | 5.91 | 209.39 | 5 | 1.8 |
| P68033 | ACTC_MOUSE | Actin. alpha cardiac muscle 1 | 41991.9 | 5.23 | 195.65 | 9 | 6.8 |
| P56480 | ATPB_MOUSE | ATP synthase subunit beta, mitochondrial | 56265.5 | 5.19 | 192.06 | 6 | 20 |
| P11103 | PARP1_MOUSE | Poly [ADP-ribose] polymerase 1 | 113028 | 9.05 | 100.2 | 2 | 0.3 |
| O35295 | PURB_MOUSE | Transcriptional activator protein Pur-beta | 33880.8 | 5.35 | 76.87 | 4 | 9.6 |
| P19324 | SERPH_MOUSE | Serpin H1 | 46560.2 | 8.9 | 69.96 | 2 | 9.4 |
| P23475 | KU70_MOUSE | ATP-dependent DNA helicase 2 subunit 1 | 69440.9 | 6.35 | 66.58 | 2 | 5.3 |
| P50543 | S10AB_MOUSE | Protein S100-A11 | 11075.5 | 5.28 | 64.81 | 1 | 15.5 |
| P27641 | KU86_MOUSE | ATP-dependent DNA helicase 2 subunit 2 | 83004 | 5.04 | 59.15 | 3 | 6.7 |
| Q99K48 | NONO_MOUSE | p54^nrb^/NONO Non-POU domain-containing octamer-binding protein | 54506.4 | 9.01 | 57.77 | 1 | 1 |
| Q03265 | ATPA_MOUSE | ATP synthase subunit alpha, mitochondrial | 59715.6 | 9.22 | 55.43 | 2 | 6.5 |
| Q569Z6 | TR150_MOUSE | Thyroid hormone receptor-associated protein 3 | 108114 | 10.17 | 49.81 | 5 | 5.6 |

E: BIA-MS data: total C2C12 extract proteins bound to the CMV promoter DNA.

| AC | ID | Description | MW | pI | Score | Peptides | Protein Coverage (%) |
| --- | --- | --- | --- | --- | --- | --- | --- |
| P21428 | ACTA_MOUSE | Actin alpha skeletal muscle | 42023.9 | 5.23 | 266.7 | 14 | 38.7 |
| P3476 | ATP-MOUSE | ATP5B ATP synthetase subunit beta mitochandrial | 41981.81 | 5.23 | 270.48 | 15 | 10.1 |
| P29744 | SSBP1_MOUSE | Single-stranded DNA-binding protein, mitochondrial | 17249 | 9.59 | 204.56 | 5 | 32.5 |
| P479743 | POTEE_MOUSE | Isoform 1 of POTE ankyrin domain family membre E | 121286 | 5.83 | 182.01 | 7 | 8 |
| P784154 | HSPD1_MOUSE | Heat shock protein, mitochondrial | 26657.41 | 9.68 | 112.42 | 6 | 11.4 |
| P7047 | S100-A8_MOUSE | S100A8 protein S100-A8 | 10827.7 | 6.51 | 167.34 | 3 | 6.8 |
| P3269 | ACTBL2_MOUSE | Beta actin-like protein 2 | 41976 | 5.39 | 148.72 | 6 | 16.8 |
| P14230 | C1QBP_MOUSE | Complement composent 1 Q subcomponent-binding protein mitochondrial | 31342.6 | 19.9 | 143.06 | 2 | 19.9 |
| P27462 | S100-A9_MOUSE | S100A9 protein | 13233.5 | 5.71 | 139.28 | 6 | 43 |
| P3935 | HT2H2BE_MOUSE | Histone H2B type 2-E | 13911.6 | 10.31 | 66.47 | 4 | 33.3 |
| P7188 | SLC25A5_MOUSE | ADP/ATP translocase2 | 32874.2 | 9.7 | 48.84 | 3 | 9.7 |
| P291467 | SLC25A6_MOUSE | ADP/ATP translocase3 | 32845.2 | 9.76 | 48.84 | 3 | 9.7 |
| P440493 | ATP5A1_MOUSE | ATP5A1 ATP synthase subunit alpha mitochondrial | 59713.6 | 9.16 | 47.52 | 4 | 9.4 |
| P26272 | HT H2A_MOUSE | Histone H2A type 1-B/E | 14127 | 11.05 | 43.44 | 1 | 22.3 |
