# Supplementary figures and images for: Promoter-Dependent Translation Controlled by p54nrb and hnRNPM during Myoblast Differentiation
Source: PLoS One. 2015 Sep 2;10(9):e0136466. doi: 10.1371/journal.pone.0136466 (PMC4558007; doi:10.1371/journal.pone.0136466)

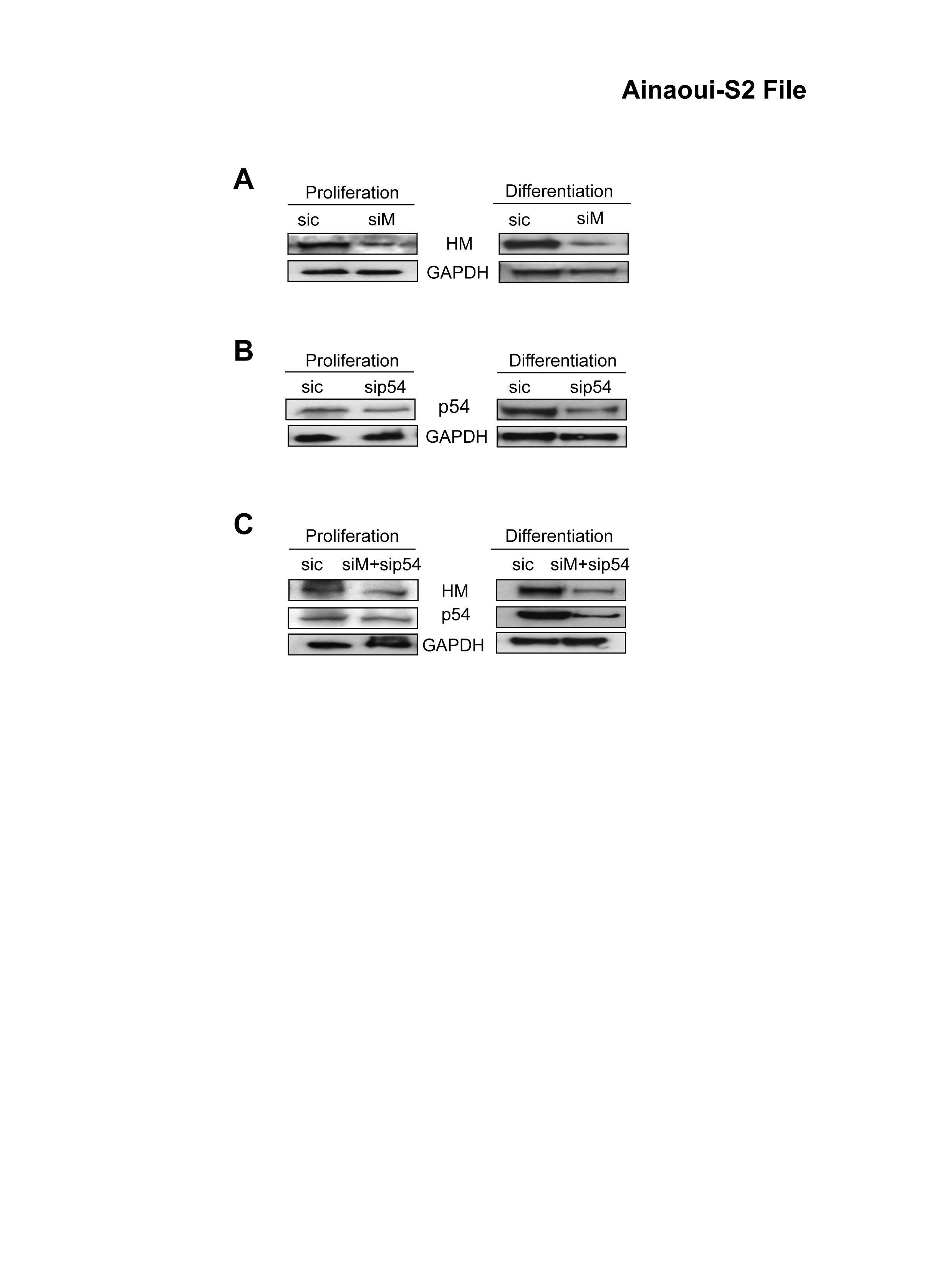

Supplement: S2 File — C2C12 cells were first transfected with bicistronic plasmids and 48h later with siRNAs targeting hnRNPM (siM), p54nrb (sip54) or control (sic). RNA levels and luciferase activities are presented in Figs 3 and 4, respectively, from C2C12 myoblasts maintained in proliferation or at day 2 of differentiation. p54nrb and hnRNPM expression was analysed by Western blot following single knockdown with siRNA siM (Fig A) or sip54 (Fig B) or double knockdown with the two siRNAs together (Fig C). (TIF) [file pone.0136466.s002.tif]

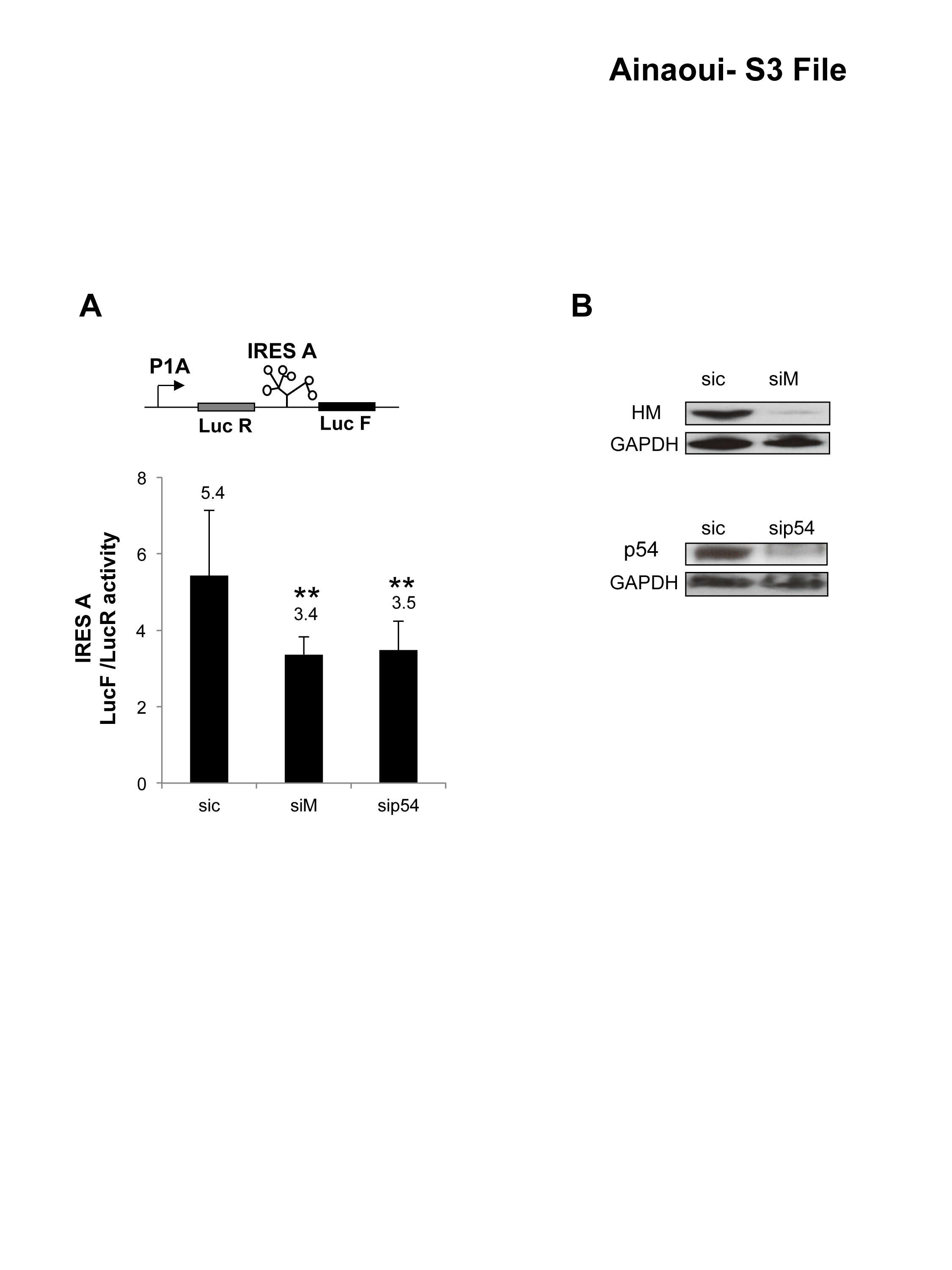

Supplement: S3 File — C2C12 cells co-transfected with bicistronic plasmids and 48h later with a siRNA against hnRNPM (siM), p54nrb (sip54) or siRNA control (sic). SiM and siP54 corresponded to sequences different from that of the siRNA smartpools used in Fig 4. (Fig A) Ratio of the luciferase activities measured as in Fig 4, two days after siRNA transfection, from differentiating C2C12 myoblasts (day 2). (Fig B) The knockdown was checked by Western blot as in S2 File. (TIF) [file pone.0136466.s003.tif]
