# Supplementary material for: Promoter-Dependent Translation Controlled by p54nrb and hnRNPM during Myoblast Differentiation
Source: PLoS One. 2015 Sep 2;10(9):e0136466. doi: 10.1371/journal.pone.0136466 (PMC4558007; doi:10.1371/journal.pone.0136466)
Supplement: S4 File — (Table A) C2C12 cells were transfected with bicistronic plasmids containing the FGF1 promoter and IRES (see Fig 6) and with siRNA siM, sip54 or sic. (Table B) C2C12 cells were transfected with siRNA siM, sip54 or sic and 24h later with bicistronic mRNA containing the FGF1 IRES (see Fig 6). mRNAs were transcribed in vitro, capped and polyadenylated, as described in Mat. & Meth. For Tables A and B, firefly and renilla luciferase activities were measured. Values are presented as well as the LucF/LucR (F/R) ratio representing the IRES activity. Experiments were performed in biological triplicates and repeated three times. The Student test was used (mean +- standard deviation). (DOC) [file pone.0136466.s004.doc]

**Ainaoui et al, S4 file.**

**A: hnRNPM and p54 knockdown after DNA transfection**: FGF1 IRES

| **Bicistronic vector**  **pP1A-R1AL2**  **(FGF1 prA**  **+ IRES A)** | **Firefly Luciferase**  **activity** | **Renilla Luciferase**  **activity** | **F/R ratio**  **(IRES activity)** |
| --- | --- | --- | --- |
| **Proliferation** |  |  |  |
| **siC** | 2672600 (±342846) | 9420 (±726) | 283 (±58) |
| **siM** | 1796600 (±154452) | 9690 (±137) | 185 (±14) |
| **sip54** | 1601300 (±46364) | 9735 (±626) | 164 (±14) |
| **Differentiation** |  |  |  |
| **siC** | 10356575 (±668574) | 17698 (±1898) | 585 (±123) |
| **siM** | 44860 (±2692) | 2875 (±356) | 15 (±2.2) |
| **sip54** | 42060 (±911) | 2576 (±297) | 16 (±0.8) |

**B: hnRNPM and p54 knockdown after RNA transfection**: FGF1 IRES

| **Bicistronic mRNA**  **R1AL2**  **(FGF1 IRES)** | **Firefly Luciferase** | **Renilla Luciferase** | **F/R ratio** |
| --- | --- | --- | --- |
| **Proliferation** |  |  |  |
| **siC** | 2770 (±29) | 4082 (±167) | 0.68 (±0.04) |
| **siM** | 2508 (±91) | 3860 (±262) | 0.65 (±0.03) |
| **sip54** | 2770 (±100) | 3847 (±243) | 0.72 (±0.01) |
| **Differentiation** |  |  |  |
| **siC** | 1356 (±17) | 2455 (±34) | 0.55 (±0.004) |
| **siM** | 1338 (±31) | 2417 (±223) | 0.55 (±0.05) |
| **sip54** | 1385 (±50) | 1990 (±159) | 0.70 (±0.05) |
